# Structural basis for a degenerate tRNA identity code and the evolution of bimodal specificity in human mitochondrial tRNA recognition

**DOI:** 10.1038/s41467-023-40354-2

**Published:** 2023-08-09

**Authors:** Bernhard Kuhle, Marscha Hirschi, Lili K. Doerfel, Gabriel C. Lander, Paul Schimmel

**Affiliations:** 1 Department of Molecular Medicine, The Scripps Research Institute, La Jolla, CA 92037 USA; 2 Department of Cellular Biochemistry, University Medical Center Göttingen, 37073 Göttingen, Germany; 3 Department of Integrative Structural and Computational Biology, The Scripps Research Institute, La Jolla, CA 92121 USA; 4 The Scripps Florida Research Institute at the University of Florida, Jupiter, FL 33458 USA

**Keywords:** tRNAs, Cryoelectron microscopy, RNA metabolism, RNA, Mitochondria

## Abstract

Animal mitochondrial gene expression relies on specific interactions between nuclear-encoded aminoacyl-tRNA synthetases and mitochondria-encoded tRNAs. Their evolution involves an antagonistic interplay between strong mutation pressure on mtRNAs and selection pressure to maintain their essential function. To understand the molecular consequences of this interplay, we analyze the human mitochondrial serylation system, in which one synthetase charges two highly divergent mtRNA^Ser^ isoacceptors. We present the cryo-EM structure of human mSerRS in complex with mtRNA^Ser(UGA)^, and perform a structural and functional comparison with the mSerRS-mtRNA^Ser(GCU)^ complex. We find that despite their common function, mtRNA^Ser(UGA)^ and mtRNA^Ser(GCU)^ show no constrain to converge on shared structural or sequence identity motifs for recognition by mSerRS. Instead, mSerRS evolved a bimodal readout mechanism, whereby a single protein surface recognizes degenerate identity features specific to each mtRNA^Ser^. Our results show how the mutational erosion of mtRNAs drove a remarkable innovation of intermolecular specificity rules, with multiple evolutionary pathways leading to functionally equivalent outcomes.

## Abstract (web summary)

Aminoacyl-tRNA synthetases catalyze the ligation of amino acids to their cognate tRNAs. Here the authors report the cryo-EM structure of a human mitochondrial seryl-tRNA synthetase•mtRNA^Ser^ complex showing how strong mutation pressure on mtRNA genes drove a rewiring of intermolecular recognition rules.

## Introduction

Many essential cellular functions depend on highly specific intermolecular interactions. The defining structural and sequence features of such fitness-related traits are usually under strong selective pressure to remove mutations that would otherwise disrupt the complementarity between interacting partners or create spurious crosstalk with non-cognate systems. However, under certain conditions the efficiency of natural selection to remove deleterious mutations is reduced. In particular, small and asexually reproducing populations, such as obligate intracellular microbial parasites and endosymbionts, are vulnerable to the accumulation of deleterious alleles by mutation and random drift^1–4^. Under such conditions, an evolutionary process known as ‘Muller’s ratchet’ leads to a steady accrual of slightly deleterious mutations, posing the risk of an irreversible erosion of sequence information over time^5,6^. Animals, including humans, are subject to the risks associated with Muller’s ratchet through their asexually reproducing mitochondria^3,7–9^. Here, we use the human mitochondrial genetic code expression machinery as a model to understand how fitness-related cellular traits that rely on highly specific intermolecular recognition, evolve at the molecular level to maintain long-term functional stability in the face of mutational disruption.

As one of the most fundamental steps in cellular information processing, the accuracy of genetic code expression depends on a network of highly specific interactions between aminoacyl-tRNA synthetases (aaRSs) and tRNAs^10–12^. In canonical (i.e. cytosolic) gene expression, this specificity relies primarily on sequence-based identity information in the tRNAs in the form of highly conserved nucleotides, termed ‘identity elements’, and their spatial distribution within the L-shaped structural scaffold that is shared by all cytosolic tRNAs^10,11,13^. Together, they constitute an ‘operational tRNA identity code’ for aaRSs to discriminate cognate from non-cognate tRNAs^14^. Prominent examples include the canonical alanylation and serylation systems from bacteria to the eukaryote cytoplasm, in which tRNA^Ala^ and tRNA^Ser^ are respectively specified by a single G3:U70 wobble pair in the acceptor stem^15,16^ and a long variable arm (V-arm)^17^, embedded into the structural scaffold of the canonical tRNA. The high degree of evolutionary conservation of structural and identity-determining sequence information in cytosolic tRNAs across widely divergent taxa implies that natural selection is usually successful in removing deleterious mutations to maintain high molecular specificity, reflecting the close relationship between genetic code expression and organismal fitness.

These principles of canonical aaRS-tRNA complementarity are challenged in the genetic code expression machineries of animal mitochondria, where high mutation rates and inefficient selection have led to an unparalleled mutational erosion of mitochondrially encoded tRNA genes^18–20^. In addition to the accumulation of weak base-pairs and mismatches in stem-regions, reduced loop sizes and even deletions of entire structural domains^20–22^, animal mtRNAs experienced a widespread loss of conserved canonical structure and sequence identity elements^19,23–29^. Despite this mutational erosion, mtRNAs and their recognition by mt-aaRSs are indispensable for mitochondrial function and cellular energy homeostasis, as demonstrated by the strong association of mtRNA and mt-aaRS mutations with a wide range of human pathologies^20,30–32^. How animal mitochondrial aaRS-tRNA interactions evolved to maintain specificity despite the erosive divergence of canonical sequence and structural features is not understood.

In this study, we focus on the mitochondrial serylation system, in which a single nuclear-encoded enzyme, mSerRS, charges two distinct mitochondria-encoded mtRNA^Ser^ isoacceptors. Both, mtRNA^Ser(GCU)^ and mtRNA^Ser(UGA)^ have lost the identity-defining long V-arm of the canonical cytosolic tRNA^Ser^, and each possesses an unusual secondary structure^19,33–35^: While mtRNA^Ser(UGA)^ adopts a non-canonical cloverleaf fold with extended anticodon stem^34,36,37^, mtRNA^Ser(GCU)^ lacks the entire D-arm^33–35^, making it the shortest and most degenerated of all human mtRNAs. To understand the molecular basis for mtRNA^Ser(GCU)^ recognition, we previously determined the structure of the human mSerRS-mtRNA^Ser(GCU)^ complex^35^. This analysis revealed a divergent recognition mechanism in which unique structural features of the Y-shaped mtRNA^Ser(GCU)^ serve as idiosyncratic identity elements for mSerRS. However, this raises the question of how mSerRS could likewise recognize the other, more canonical mtRNA^Ser(UGA)^ isoacceptor^23,24^. Here, we used single-particle cryo-EM to determine the structure of human mSerRS in complex with mtRNA^Ser(UGA)^. Combined with biochemical and evolutionary analyses, the two complexes form the basis for a detailed molecular structural and functional comparison between mtRNA^Ser(UGA)^ and mtRNA^Ser(GCU)^ and their interactions with mSerRS. Our results provide insight into the evolution and functional properties of an essential cellular machinery exposed to high mutation pressure and random genetic drift. They highlight an underlying evolutionary dynamic in which biological innovation is driven by the pressure to maintain indispensable molecular functions under conditions of mutational disruption.

## Results

### Structure of the mSerRS-mtRNA^Ser(UGA)^ complex

**Fig. 1:**
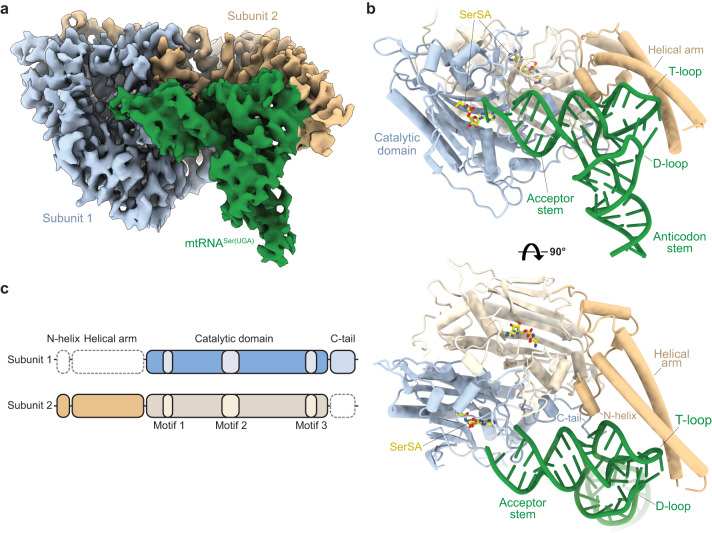
Structure of human mSerRS bound to SerSA and mtRNASer(UGA). **a** Segmented cryo-EM map of the mSerRS-mtRNA^Ser(UGA)^ complex. mSerRS is shown in light blue (subunit 1) and wheat (subunit 2), the tRNA is shown in green. **b** Atomic model of the mSerRS-mtRNA^Ser(UGA)^ complex in cartoon presentation. mSerRS and the tRNA are colored as in **a**. The SerSA ligand is shown as yellow sticks. **c** Domain presentation of mSerRS. Dashed lines indicate structural domains not defined in subunits 1 (top) and 2 (bottom) in the cryo-EM reconstruction of mtRNA^Ser(UGA)^-bound mSerRS shown in **a** and **b**.

To understand the molecular basis for mtRNA^Ser(UGA)^ recognition in human mitochondria, we reconstituted the ternary complex between human mSerRS, the seryl-adenylate analogue 5’-O-[N-(L-seryl)sulfamoyl] adenosine (SerSA), and a stabilized mtRNA^Ser(UGA)^ transcript (see Supplementary Fig. 1 and Methods for details), and determined its structure at 3.6 Å resolution using single-particle cryo-EM (Fig. 1, Supplementary Fig. 2 and 3a, and Supplementary Table 1). The reconstruction shows a well-defined density for a single tRNA bound to the synthetase dimer (Fig. 1a). The density was of sufficient quality to model ~87% of the mtRNA^Ser(UGA)^ sequence, including the acceptor-stem-T-arm domain, D-arm, and two-thirds of the anticodon stem (Fig. 1b). Docked across the mSerRS dimer interface, mtRNA^Ser(UGA)^ establishes interfaces with the active site entrance of subunit 1, the ‘C-tail’ and ‘N-helix’ of subunits 1 and 2, respectively, and the helical arm of subunit 2. Both active sites are occupied by a SerSA ligand, but only one of the two tRNA-binding sites is occupied by a tRNA. The helical arm, N-helix, and most of the C-tail that together comprise part of the second tRNA binding site, are not resolved in the EM density, likely due to flexibility (Fig. 1b, c). Analysis of the mSerRS-mtRNA^Ser(UGA)^ complex by mass photometry further supports the 1:2 stoichiometry of tRNA to mSerRS (Supplementary Fig. 3b), suggesting that the human mSerRS dimer preferably binds a single mtRNA in solution. This asymmetry in the mSerRS-mtRNA^Ser(UGA)^ complex, which is also observed for the mtRNA^Ser(GCU)^ isoacceptor^35^, suggests that its two tRNA binding sites are not equivalent once one is occupied, similar to canonical SerRSs^38–40^.

### The non-canonical structure of mtRNA^Ser(UGA)^

**Fig. 2:**
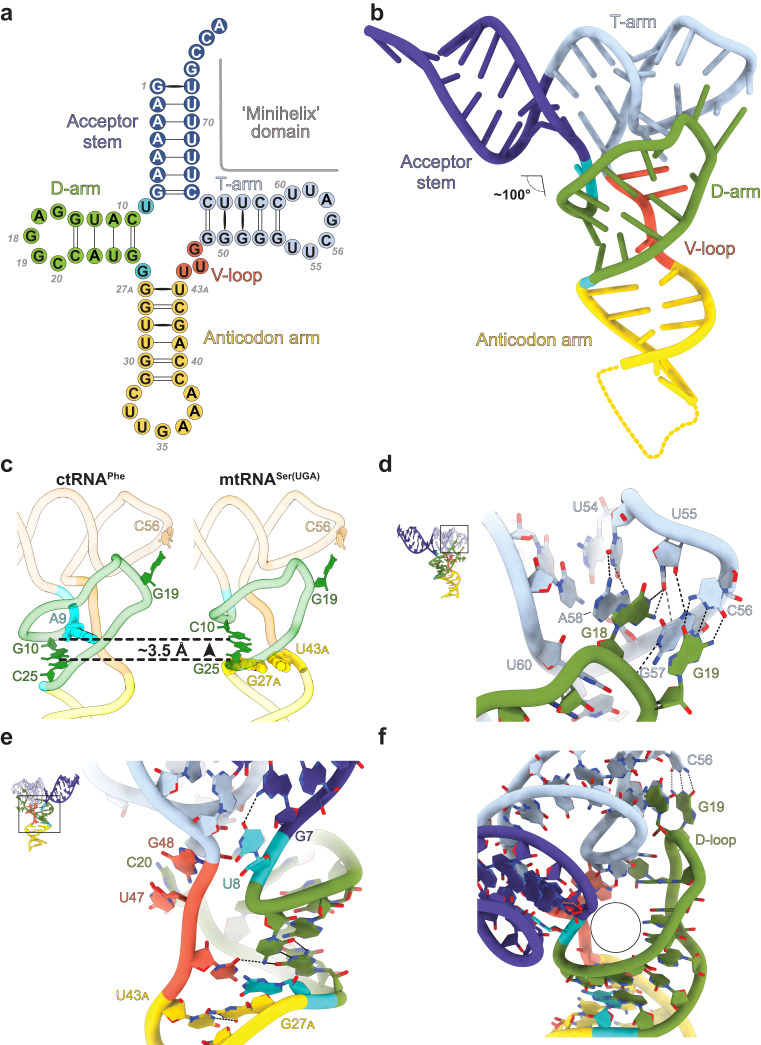
The structure of mtRNA^Ser(UGA)^. **a** Secondary structure diagram of mtRNA^Ser(UGA)^. **b** Tertiary structure of mtRNA^Ser(UGA)^ as bound to mSerRS. The portion of the anticodon arm missing in the reconstruction is indicated as a dashed yellow line. **c** Comparison of the tRNA core region in human mtRNA^Ser(UGA)^ (right) and the canonical *S. cerevisiae* ctRNA^Phe^ (left; PDB 4TRA)^75^ centered on the D-arm (green). Deletion of A9 (cyan spheres in tRNA^Phe^) results in an upward shift of the first D-arm base-pair (green sticks) in mtRNA^Ser(UGA)^ by one base-plane (indicated by dashed lines), allowing canonical interactions between the shortened D-loop and T-loop. The upward-shift of the D-arm by one base-plane is compensated in mtRNA^Ser(UGA)^ by the introduction of the additional anticodon stem base-pair G27_A_:U43_A_ (yellow spheres). **d** Details of the D-loop-T-loop tertiary interactions forming the elbow region in mtRNA^Ser(UGA)^. The T-loop of mature human mtRNA^Ser(UGA)^ contains modifications in positions U54 (m^5^U), U55 (ψ), and A58 (m^1^A)^41^. Colored as in **a** and **b**. **e** Details of the tertiary core region in mtRNA^Ser(UGA)^, centered on the V-loop. Colored as in **a** and **b**. **f** Close-up view of the hollow core structure of mtRNA^Ser(UGA)^ (circle), lacking most tertiary interactions known from canonical tRNAs.

The mSerRS-bound mtRNA^Ser(UGA)^ adopts a non-canonical L-shaped fold, with acceptor-stem-T-arm and anticodon-arm-D-arm domains arranged at an inter-domain angle of ~100° (Fig. 2a, b). The T-loop of mtRNA^Ser(UGA)^ is canonical in sequence and structure, whereas its D-loop is significantly reduced in size to five instead of the canonical eight nucleotides. In canonical tRNAs, this would prevent the D-loop from forming interactions with the T-loop. However, in mtRNA^Ser(UGA)^ the additional deletion of A9 in the linker between acceptor- and D-stem results in a shift of the entire D-stem by ~3.5 Å (one stacking plane) toward the T-arm (Fig. 2c, d). This allows G18 and G19 of the shortened D-loop to form a canonical elbow structure with the T-loop, consistent with earlier chemical probing and NMR data^36,37^. In the mature human mtRNA^Ser(UGA)^, the T-loop is post-transcriptionally modified in positions U54 (m^5^U), U55 (ψ), and A58 (m^1^A)^41^. All three of these modifications are also found in cytosolic tRNAs, where they are thought to promote and stabilize the canonical T-loop fold and its tertiary interactions with the D-loop^36,42,43^. Notably, outside of the elbow region, mtRNA^Ser(UGA)^ has lost virtually all tertiary core interactions known from canonical tRNAs, and only few alternative, unconventional interactions are potentially formed by the flipped-out U8 with G7 (instead of the canonical U8:A14 pair), and by G48 (V-loop), which stacks with U59 (T-loop) and is positioned for hydrogen-bonding with C20 (D-loop) (Fig. 2e). The consequence is a hollow tRNA core structure (Fig. 2f), which may explain the reduced thermal stability of mtRNA^Ser(UGA)^ when compared to canonical tRNAs^36^. Since deletion of A9 reduces the tRNA core dimensions, the introduction of an additional base-pair (G27_A_:U43_A_) into the anticodon stem is required to restore normal dimensions of the anticodon-D-arm domain (Fig. 2a, c)^44^.

Interestingly, several of the unusual structural features of mtRNA^Ser(UGA)^ are also found in the non-canonical tRNA^Pyl^ found in various prokaryotes^45,46^, which include the reduced D-loop size, deletion of A9, flipped-out U8, and the extension of the anticodon stem (Supplementary Fig. 4). The distinct evolutionary origins of mtRNA^Ser(UGA)^ and tRNA^Pyl^ imply that their shared non-canonical features are the result of convergent evolution.

### One identity in two distinct mtRNA^Ser^ structures

**Fig. 3:**
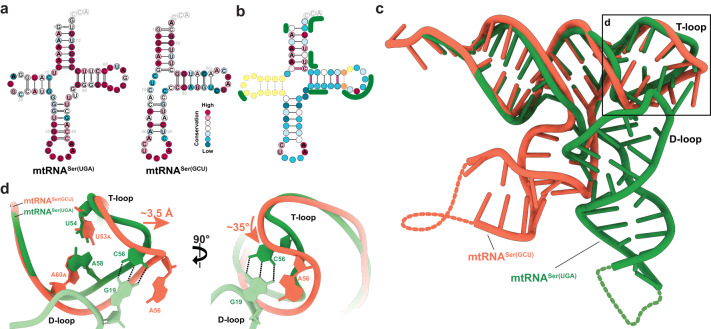
Sequence and structure comparison between mtRNA^Ser(UGA)^ and mtRNA^Ser(GCU)^. **a** Sequence conservation in mtRNA^Ser(UGA)^ (left) and mtRNA^Ser(GCU)^ (right) across 41 primate species plotted onto the secondary structures of human mtRNA^Ser^ variants. Conservation scores reach from 100% (red) to <50% (teal). **b** Sequence conservation between primate mtRNA^Ser(UGA)^ and mtRNA^Ser(GCU)^ variants. Yellow indicates nucleotides present only in mtRNA^Ser(UGA)^, orange indicates nucleotides present only in mtRNA^Ser(GCU)^. Nucleotide identities are given if positions are >90% conserved and shared between both human tRNA^Ser^ isoacceptors. Green lines indicate the interfaces of both tRNAs with mSerRS. **c** Superposition of the tertiary structures of mtRNA^Ser(UGA)^ (green) and mtRNA^Ser(GCU)^ (tomato; PDB 7U2A)^35^. The superposition is based on the acceptor-stem-T-stem regions of both tRNAs. The region enlarged in **d** is indicated. **d** Close-up view of the superimposed elbow regions from mtRNA^Ser(UGA)^ (green) and mtRNA^Ser(GCU)^ (tomato), showing the shift and rotation of A56 in mtRNA^Ser(GCU)^ relative to the homologous C56 in mtRNA^Ser(UGA)^ as a consequence of the additional T-stem pair U53_A_:A60_A_ (see also **b**). Dashed lines indicate the H-bonds of the tertiary G19:C56 base pair that is found in all canonical tRNAs, but absent from mtRNA^Ser(GCU)^.

It has long been noted that mammalian mtRNA^Ser(UGA)^ and its isoacceptor mtRNA^Ser(GCU)^ have markedly distinct secondary structures, with one folding into a near-canonical cloverleaf, while the other adopts a non-canonical fold lacking the D-arm^20,33,47^. This raises the question of whether the two isoaccepting tRNAs share any conserved sequence or structural features that could serve as common identity-determining recognition elements for mSerRS^47^. Sequence analysis shows that both mtRNA^Ser^ isoacceptors are poorly conserved even among closely related species (Fig. 3a), reflecting the high sequence evolution rates among animal mtRNAs. Across 41 primate species (based on sequences currently deposited in the tRNAdb http://trna.bioinf.uni-leipzig.de/), only ~43% of positions (30 out of 69) are conserved in mtRNA^Ser(UGA)^ (Fig. 3a). Conservation is even further reduced to ~31% (18 out of 59) in mtRNA^Ser(GCU)^ (Fig. 3a). In both cases, the highly conserved sites are located around the anticodon loop, the acceptor stem, and the T-loop. Surprisingly, only six of these positions are also conserved across primate mtRNA^Ser(UGA)^ and mtRNA^Ser(GCU)^ variants (Fig. 3b). These include three positions in the acceptor stem (A4, A6, and U71) and U55 in the T-loop, the only position conserved in the T-arms of both isoacceptors. Interestingly, the overall highest degree of sequence similarity is found in the four base-pairs at the base of the acceptor stem. All other regions show significant variability, most notably in the discriminator base, the first base-pair of the acceptor stem, the T-stem, and T-loop (except U55). The divergence of the T-arm is particularly notable, as it also includes a mtRNA^Ser(GCU)^ isoacceptor-specific insertion of an additional base-pair into the T-stem and simultaneous deletion of one T-loop nucleotide relative to mtRNA^Ser(UGA)^ (Fig. 3b).

Superposition of the mtRNA^Ser(UGA)^ and mtRNA^Ser(GCU)^ structures shows that this lack of sequence identity translates into distinct tertiary structures, as the two tRNAs show virtually no overlap outside of the coaxially stacked helical portions of the acceptor- and T-stems (Fig. 3c). Particularly notable is the divergence of the elbow regions (Fig. 3c, d). In the UGA isoacceptor, the elbow region maintains a nearly canonical fold, with the nucleobases A58, G18, G57, and G19 forming a continuous, intercalating stack between the canonical T-loop and near-canonical D-loop (Fig. 2d and Supplementary Fig. 5). The G19:C56 Watson-Crick base-pair forms the elbow’s distal end, presenting a flat hydrophobic surface to the solvent. In stark contrast, the GCU isoacceptor has lost virtually all canonical structural and sequence features and contains no tertiary core^35^. In the absence of a D-loop and any tertiary interactions between the vestigial ‘D-replacement loop’ and the T-arm, the ‘T-loop^Ser(GCU)^’ evolved a unique, self-contained loop topology that shares little sequence and structural homology with its near-canonical counterpart (Supplementary Fig. 5)^35^ and is not post-transcriptionally modified^41^. Most notably, the distal G19:C56 tertiary pair is replaced in mtRNA^Ser(GCU)^ by an unpaired A56, which, due to the insertion of an entire additional stacking plane into the ‘T-arm^Ser(GCU)^’, is shifted outward by ~3.5 Å and rotated by ~36° relative to the corresponding C56 in mtRNA^Ser(UGA)^ (Fig. 3d and Supplementary Fig. 5).

In summary, our results reveal a striking lack of sequence or structural identity between the two human mitochondrial serine isoacceptors. Indeed, a wider cross-comparison of primary, secondary, and available tertiary structures of the 22 human mitochondrial tRNAs shows that mtRNA^Ser(UGA)^ and mtRNA^Ser(GCU)^ lie on opposite sides of the wide structural spectrum of mtRNAs (Supplementary Fig. 6), highlighting the unusual degree of divergence between two tRNAs that share a common function in mitochondrial translation.

### Bimodal recognition of mtRNA^Ser(UGA)^ and mtRNA^Ser(GCU)^ by mSerRS

In the canonical bacterial and eukaryote cytoplasmic aminoacylation systems, the major identity-defining elements are shared between tRNAs belonging to the same isoacceptor family^10,11^. As one of most prominent examples for this unimodal recognition principle, canonical tRNA^Ser^ from bacteria to the eukaryote cytoplasm are recognized by SerRS by their extended V-arm (Supplementary Fig. 7)^17^. Both mitochondrial tRNA^Ser^ isoacceptors have lost the long V-arm of canonical tRNA^Ser^ (Fig. 3a), and, like their canonical counterparts, are not recognized through their anticodons^34,47,48^. How then does a single mSerRS maintain intermolecular specificity for two mtRNA^Ser^ substrates that lack shared structural or conserved sequence identity features?

**Fig. 4:**
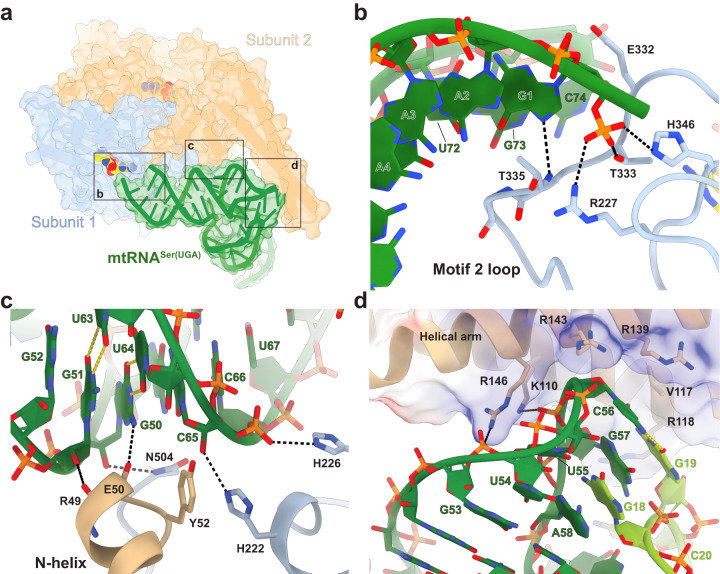
Binding interfaces between mtRNA^Ser(UGA)^ and mSerRS. **a** Overview of the mSerRS-mtRNA^Ser(UGA)^ complex with regions enlarged in **b,** **c**, and **d** indicated. **b** Close-up view of the interactions between the acceptor arm of mtRNA^Ser(UGA)^ (green) and the motif 2 loop (blue) at the entrance to the active site. **c**. Close-up view of the interactions of the T-stem (green) with the N-helix (wheat) and C-tail (blue) of mSerRS. **d** Close-up view of the interface between the T-loop (green) and the helical arm (wheat). Side chains of mSerRS are shown as sticks. Arg118, Arg139, and Arg143 are not resolved in the EM map and shown as their energetically most favorable rotamers. The electrostatic surface potential of mSerRS is shown transparently as a three-color gradient scheme from −5 to +5 kcal mol^−1^ e^−1^ (red, negative; white, neutral; blue, positive).

**Fig. 5:**
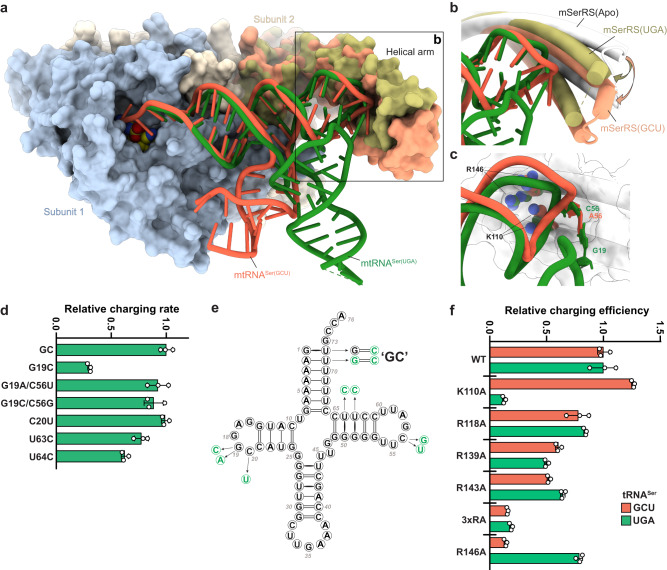
Comparison of interactions of mSerRS with mtRNA^Ser(UGA)^ and mtRNA^Ser(GCU)^. **a** Superposition of the mSerRS-mtRNA^Ser(UGA)^ and mSerRS-mtRNA^Ser(GCU)^ complexes. The body of mSerRS is shown in light blue (subunit 1) and wheat (subunit 2), the helical arms are colored differentially in light green (UGA complex) and light tomato (GCU complex), respectively. mtRNA^Ser(UGA)^ is colored in green, mtRNA^Ser(GCU)^ in tomato (PDB 7U2A)^35^. The superposition is based on the catalytic core domains of the mSerRS dimer. **b** Close-up view of the helical arm-elbow interface in both complexes as shown in **a**, highlighting the conformational changes in the helical arm induced by tRNA binding (arrows). The helical arm of the free mSerRS (PDB 7TZB) is shown in white. **c** Close-up view of the helical arm-elbow interface in a superposition based on the helical arm (shown white cartoon with transparent surface). The side chains of Lys110 (K110) and Arg146 (R146) are shown as spheres and colored green for the mtRNA^Ser(UGA)^ complex and tomato for the mtRNA^Ser(GCU)^ complex. **d** Summary of charging experiments for mtRNA^Ser(UGA)^ mutants by wild-type mSerRS. **e** Summary of mtRNA^Ser(UGA)^ mutants used in **d**. ‘GC’ denotes stabilized ‘wild-type’ tRNA (see Supplementary Fig. 1 and Methods). **f** Charging kinetics for mSerRS variants on mtRNA^Ser(UGA)^ (green) and mtRNA^Ser(GCU)^ (tomato; data taken from Ref. ^35^). 3xRA denotes the R118A, R139A, R143A triple mutant. Data are presented as mean values+/-SD from triplicate experiments. Individual data points are shown as open circles. Source data are provided as a Source Data file. See also Supplementary Tables 3 and 4.

The comparison of the two human mSerRS-mtRNA^Ser^ complexes shows that mSerRS binds both mtRNA^Ser^ isoacceptors using virtually identical binding surfaces (Figs. 4 and 5). Like the GCU isoacceptor, mtRNA^Ser(UGA)^ docks onto the mSerRS dimer via three major interfaces (Fig. 4a): The tRNA acceptor end is positioned at the active site entrance of subunit 1 (Fig. 4b); the T-stem is packed against a combined interface formed by the ‘C-tail’ and ‘N-helix’ of subunits 1 and 2, respectively (Fig. 4c); and the elbow region contacts the helical arm of subunit 2 (Fig. 4d). Neither the D-stem, anticodon arm, or the reduced three-nucleotide V-loop (_45_UUG_47_), a vestige of the identity-determining long V-arm of canonical tRNA^Ser^, contribute to the interaction. Thus, mtRNA^Ser(UGA)^ is bound almost exclusively through its acceptor-T-arm (‘minihelix’) domain, similar to the GCU isoacceptor^35^. A notable exception is the additional contact by mSerRS with G19 from the D-loop of mtRNA^Ser(UGA)^ (Fig. 4d), which is absent in mtRNA^Ser(GCU)^ (Fig. 3).

To bind mtRNA^Ser(UGA)^, mSerRS uses 23 amino acid residues that are nearly identical to those involved in GCU isoacceptor binding. Most contacts with mtRNA^Ser(UGA)^ are with the phosphate-sugar backbone, distributed along the entire acceptor-T-arm domain. As for the mtRNA^Ser(GCU)^ isoacceptor^35^, mtRNA^Ser(UGA)^ binding induces a large conformational rearrangement in the motif 2 loop in the entrance to the active site, which inserts residues 334-340 into the major groove of the acceptor stem and forms putative base-specific interactions with C74 of the CCA-end, ‘discriminator base’ (G73), and the first acceptor-stem pair G1:U72. However, neither G73 nor the G1:U72 base-pair are conserved among primate mtRNA^Ser(UGA)^ sequences and differ even between the two human mtRNA^Ser^ isoacceptors (Fig. 3a, b), and substitution of the G1:U72 wobble pair by G1:C72 has no negative effect on aminoacylation by mSerRS (Supplementary Fig. 1), consistent with previous results from the human and bovine mitochondrial serylation systems^23,35,49^. Interestingly, the A2:U71 pair is the only acceptor-stem pair that is nearly universally conserved among primate mtRNA^Ser^ variants (Fig. 3a, b). However, as observed in the GCU isoacceptor^35^, A2:U71 of human mtRNA^Ser(UGA)^ is not in direct contact with mSerRS (Fig. 4b), and its substitution with G2:C71 has no adverse effect on aminoacylation (Supplementary Fig. 1).

The second major mtRNA^Ser(UGA)^ binding interface is formed between the mSerRS-specific N-helix and C-tail and the minor groove side of the tRNA’s T-stem (Fig. 4c). Within this interface, nearly all contacts are made with the tRNA’s phosphate-sugar backbone. Only one base-specific H-bond is formed by the backbone amide oxygen of Glu50 to the exocyclic 2-amino group of G50 (~ 3 Å), which is unpaired and shifted to the minor groove side due to the wobble geometry of G50:U64. However, the G50:U64 pair is only partially conserved in primate mtRNA^Ser(UGA)^ sequences (~ 70%) and frequently replaced by G:C or A:U Watson-Crick pairs (Fig. 3a). Interestingly, human mtRNA^Ser(UGA)^ contains a second wobble pair (G51:U63) adjacent to G50:U64, and at least one of the two wobble pairs is found in >95% of mammalian mtRNA^Ser(UGA)^ sequences. Although G51:U63 is not in direct contact with the synthetase, mutations in both G:U pairs affect mtRNA^Ser(UGA)^ aminoacylation (Fig. 5d), suggesting that their contribution is indirect, possibly by a local distortion or/and increased flexibility in the T-stem helix. Notably, the other isoacceptor, mtRNA^Ser(GCU)^, contains two Watson-Crick pairs, C50:G64 and A51:U63, in the corresponding interface (Fig. 3a), neither of which is important for aminoacylation^35^. This suggests that the contribution of the G:U wobble pairs in the T-stem interface is idiosyncratic to mtRNA^Ser(UGA)^ recognition, and may provide a mechanistic rationale for the distinct effects the removal of the C-tail (deleting 15 residues from the C-terminus) has on the *k*_cat_ and *K*_m_ between bovine mSerRS and mtRNA^Ser(UGA)^ versus mtRNA^Ser(GCU)^.^24^ The importance of the C-tail for mtRNA^Ser(UGA)^ recognition is further highlighted by mutational studies in the bovine system, which found that a G49:U65 wobble pair at the base of the T-stem acts as a negative determinant for mSerRS and reduces non-cognate mtRNA^Gln^ misaminoacylation^23^. The structure does not provide a clear explanation for the discrimination against G49:U65, as mSerRS appears to form no direct contact with G49:C65 in mtRNA^Ser(UGA)^ (or the C49:G65 pair in mtRNA^Ser(GCU)^). However, the proximity of the side chain of Asn504 in the C-tail of mSerRS may suggest a steric incompatibility with the wobble-pair-induced shift of the exocyclic 2-amino group of G49 toward the minor groove (Fig. 4c).

The third mSerRS-mtRNA^Ser(UGA)^ interface is formed by the synthetase’s helical arm and the tRNA’s elbow region (Fig. 4d). Comparison with the free synthetase shows that mtRNA^Ser(UGA)^ binding induces a conformational rearrangement in the helical arm (Fig. 5b), which inserts the distal G19:C56 tertiary pair into a mSerRS-specific binding pocket of the helical arm framed by Lys110, Arg118, Arg139, Arg143, and Arg146 (Fig. 4d). The helical arm thereby adopts two distinct substrate-induced conformations to differentially accommodate the idiosyncratic structural requirements of mtRNA^Ser(UGA)^ and mtRNA^Ser(GCU)^ (Fig. 5b). Notably, this allows the G19:C56 pair of mtRNA^Ser(UGA)^ and the unpaired A56 of mtRNA^Ser(GCU)^ to occupy virtually identical positions at the bottom of the helical arm binding pocket (Fig. 5b, c). The G19:C56 pair in mtRNA^Ser(UGA)^ and A56 in mtRNA^Ser(GCU)^ thus seem to play analogous functional roles in the interaction with mSerRS. This is further supported by mutational analysis, which shows that the disruption of the G19:C56 tertiary interaction in the G19C mutant results in the loss of aminoacylation by mSerRS in both the bovine^23^ and the human system (Fig. 5d), similar to the effect of A56C in mtRNA^Ser(GCU)^^35^. Conversely, reintroduction of A19:U56 or C19:G56 Watson-Crick pairs restores aminoacylation activity (Fig. 5d)^23^, demonstrating that mtRNA^Ser(UGA)^ recognition is not based on the sequence identity of the G19:C56 pair, but primarily on the structural integrity of the distal elbow region. This may explain at least in part how canonical post-transcriptional modifications, including ψ55 and m^1^A58 that promote the internal folding of the T-loop and its tertiary interactions with the D-loop, increase the efficiency of bovine mtRNA^Ser(UGA)^ aminoacylation^36^. Importantly, none of the bases that are post-transcriptionally modified in mature human mtRNA^Ser(UGA)^ are in direct contact with the synthetase (Fig. 4d), arguing against a more direct role of these modifications as identity elements. Finally, the dependency of mSerRS on a canonical elbow structure to recognize mtRNA^Ser(UGA)^ supports the notion that misaminoacylation of mtRNA^Gln^ by bovine mSerRS is a result of the structural similarities of the elbow regions in both tRNAs^23^.

As in the GCU isoacceptor complex^35^, no sequence-specific interactions appear to occur between mSerRS and the mtRNA^Ser(UGA)^ elbow. Surrounding the G19:C56 binding pocket, Lys110, Arg139, Arg143, and Arg146 are all within contact distance to the phosphate backbone of the T-loop, while Arg118 lies close to C20 of the D-loop (Fig. 4d). Although individual alanine mutations of Arg118, Arg139, Arg143, and Arg146 do not have a strong impact on mtRNA^Ser(UGA)^ charging (with the strongest being a 2-fold reduction by R139A) (Fig. 5f), the R118A, R139A, and R143A triple mutation (‘3xRA’) significantly reduces charging (Fig. 5f), indicating their collective importance for tRNA binding. Interestingly, R146A, which abolishes charging on mtRNA^Ser(GCU)^, had virtually no impact on the aminoacylation of mtRNA^Ser(UGA)^ (Fig. 5f). Instead, the alanine mutation of Lys110, which is close to the backbone phosphate of G57, causes the severest decrease in mtRNA^Ser(UGA)^ aminoacylation activity (Fig. 5f), whereas it has virtually no effect on GCU isoacceptor charging. Both observations are consistent with results from the bovine mitochondrial serylation system^23,24^. Thus, Arg146 and Lys110 appear to play central and conserved roles in isoacceptor-specific recognition of mammalian mtRNA^Ser(GCU)^ and mtRNA^Ser(UGA)^, consistent with their early fixation and strong conservation in the vertebrate lineage, along with the other characteristics of bimodal mtRNA^Ser^ recognition (Supplementary Fig. 8 and 9). The molecular basis for this isoacceptor-specificity appears to lie in the local topological differences between mtRNA^Ser(GCU)^ and mtRNA^Ser(UGA)^, with the 5’ portion of the canonical ‘T-loop^Ser(UGA)^’ notably less arched and closer to the peptide backbone than in the non-canonical ‘T-loop^Ser(GCU)^’ (Fig. 5c).

Taken together, mSerRS binds both mtRNA^Ser^ isoacceptors through a single binding interface. The identity elements of mtRNA^Ser(GCU)^ and mtRNA^Ser(UGA)^ that are recognized by mSerRS are not related to each other either by sequence or by structure, resulting in two orthogonal identity sets that cannot be interconverted into each other by point mutations or transplantation of individual sub-components. As a consequence, the cognate mitochondrial synthetase appears to have evolved a bimodal recognition mechanism that uses distinct subsets of amino acid side chains and induced fit adaptation to achieve complementarity with each of its divergent mtRNA^Ser^ substrates.

## Discussion

Our preceding analysis of the human mitochondrial mSerRS-mtRNA^Ser^ recognition system addresses the question of how an individual molecular machinery evolves and maintains intermolecular specificity under conditions of strong mutation pressure and genetic drift. A central observation from this analysis is the degree of divergence of the two human mtRNA^Ser^ isoacceptors mtRNA^Ser(GCU)^ and mtRNA^Ser(UGA)^, not only from the ancestral canonical tRNA^Ser^, but from each other. Both mtRNA^Ser^ isoacceptors exhibit strong mutational erosion, which has led to the loss of highly conserved structural and sequence elements that otherwise define canonical tRNA^Ser^ variants throughout the three domains of life^17^. This divergence resulted in each mtRNA^Ser^ isoacceptor evolving idiosyncratic sequence and structural features in their core and elbow regions that are not found in other human mtRNAs. Indeed, cross-comparison shows that mtRNA^Ser(GCU)^ and mtRNA^Ser(UGA)^ lie at opposite ends of the wide structural spectrum that characterizes the 22 human mtRNAs, respectively adopting one of the most divergent and one of the most canonical folds. Thus, despite sharing a common function and interaction partners in mitochondrial gene expression, mtRNA^Ser(GCU)^ and mtRNA^Ser(UGA)^ did not converge on a common sequence or structure like their canonical counterparts, but diverged along distinct evolutionary trajectories.

**Fig. 6:**
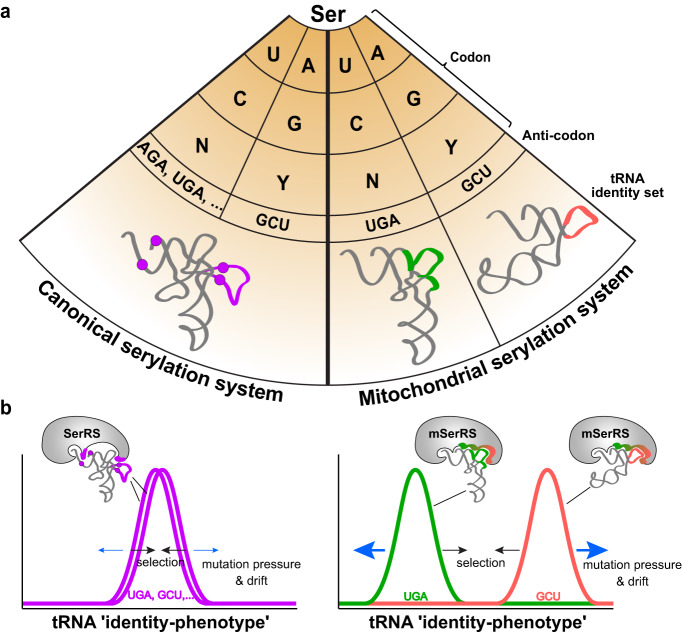
Mitochondrial tRNA^Ser^ isoacceptors use a degenerate operational tRNA identity code to specify serine. **a** Comparison of the relationship between codon identities (genetic code) and tRNA identity sets (operational tRNA identity code) for the canonical (left) and the mitochondrial (right) serylation systems. While in canonical systems, all tRNA^Ser^ isoacceptors share the same identity set (purple), human mtRNA^Ser(UGA)^ and mtRNA^Ser(GCU)^ contain distinct sets of identity elements (highlighted in green and tomato, respectively), both specifying serine. N stands for all four bases (A, G, C, U), Y stands for pyrimidine bases (C, U). **b** Schematic presentation of bimodality in human mSerRS-mtRNA^Ser^ recognition. In canonical systems (left), natural selection (black arrows) efficiently eliminates deleterious mutations, thereby maintaining a shared ‘identity-phenotype’ among all tRNA^Ser^ isoacceptors and unimodal recognition by SerRS. In animal mitochondria (right), the increased bias toward mutation pressure and drift (blue arrows) drove the mutational divergence of mtRNA^Ser(UGA)^ and mtRNA^Ser(GCU)^ toward distinct ‘identity-phenotypes’ and the evolution of a bimodal recognition mechanism by mSerRS.

The degree of divergence between isoaccepting tRNAs stands in marked contrast to the structural and sequence uniformity known from canonical cytosolic tRNAs^13,43^ and raises the question of how a single synthetase maintains complementarity with both substrates^23,24^. In contrast to the principle of unimodality found among canonical aaRS-tRNA recognition systems, human mSerRS employs a bimodal recognition mechanism, in which it uses a single binding surface and substrate-specific induced fit to specifically recognize each mtRNA^Ser^ isoacceptor by orthogonal sets of identity elements (Fig. 6). Notably, neither of the two mtRNA^Ser^ identity sets overlaps with the identity set observed in canonical SerRS-tRNA^Ser^ interactions^17^. This suggests that the divergent substrate-engagement mechanisms in mSerRS-mtRNA^Ser^ interactions are not merely the leftover vestiges of an eroded ancestral state, but rather represent genuine innovations of the animal mitochondrial gene expression machinery driven by the antagonistic interplay between strong mutation pressure acting on mtDNA-encoded tRNAs on the one hand and strong selection pressure to maintain their indispensable function on the other. This is further supported by functional data from the bovine mitochondrial serylation system^23,24^, as well as by the deep phylogenetic origin of key recognition elements in mSerRS and mtRNA^Ser^ isoacceptors, suggesting that the basic mechanism of bimodal mtRNA^Ser^ recognition is conserved across the vertebrate lineage (Supplementary Fig. 9).

The causes underlying the increased susceptibility of animal mtRNAs to mutation accumulation have been a long-standing question^7,9,18,19^. One hypothesis proposes that the elevated speed of mtRNA gene evolution is linked to a relaxation of functional constraints acting on the reduced number of mtRNAs in mitochondrial gene expression^50^. The loss and rewiring of otherwise invariant tRNA identity elements in the human mitochondrial serylation system and in other mitochondrial aminoacylation systems^19,28^, and in particular the loss of strict aminoacylation barriers between mtRNAs, such as mtRNA^Ser(UGA)^ and mtRNA^Gln^ due to similar elbow regions^23^, are consistent with this hypothesis. Moreover, it suggests that asymmetries in functional constraints, such as distinct codon frequencies (> 4-fold less AGY than UCN codons in mtDNA-encoded genes)^32,51^ and alternative tRNA processing mechanisms^52^, may facilitate the evolutionary divergence of mtRNA^Ser(UGA)^ and mtRNA^Ser(GCU)^.

An important characteristic of both mtRNA^Ser^ identity sets in the human mitochondrial serylation system is that they show virtually no sequence-specificity^35^, with no apparent requirements to maintain invariant nucleotide positions. This allows apparently unrelated sequence elements, such as A56 in mtRNA^Ser(GCU)^ and the G19:C56 pair in mtRNA^Ser(UGA)^, to play functionally equivalent roles in their interactions with mSerRS, and implies that the bimodal recognition mechanism by mSerRS allows mtRNA^Ser(UGA)^ and mtRNA^Ser(GCU)^ to evolve rapidly and essentially independently from each other, without being constrained to a single structure or sequence motif like their canonical counterparts. These observations are consistent with theoretical expectations for the divergence of intermolecular recognition motifs in scenarios where several binding partners interact with a single protein^53^, and suggest that the divergent evolution of mtRNA^Ser(UGA)^ and mtRNA^Ser(GCU)^ recognition motifs, despite selection for high specificity, may be driven primarily by the relaxation of functional constraints^50^, mutation pressure, and genetic drift^18^, rather than adaptive fine-tuning.

The bimodality of mSerRS-mtRNA^Ser^ interactions marks a fundamental shift in the relationship between tRNA identities, amino acids, and the genetic code. In canonical systems, major identity elements specifying a particular amino acid are usually shared between all isoaccepting tRNAs^10,11^. Thus, while amino acid-codon assignments (i.e. the genetic code) are often degenerate, the assignment between tRNA identity elements and amino acids (i.e. the operational tRNA identity code) is not^14,54^. Prominent examples for this non-degenerate relationship include alanine and serine. Both are encoded by multiple mRNA codons. Yet, in all canonical systems studied so far, alanine is specified by a single G3:U70 wobble pair in the acceptor stem of tRNA^Ala^ isoacceptors^15,16^, whereas the identity of canonical tRNA^Ser^ is established by its long V-arm^17^. The human mitochondrial serylation system stands in a marked contrast to this principle of unimodality, as no major identity features appear to be shared between mtRNA^Ser(GCU)^ and mtRNA^Ser(UGA)^. Thus, serine is specified in human mitochondria by a degenerate operational tRNA identity code, which is embedded into the distinct structures of mtRNA^Ser(GCU)^ and mtRNA^Ser(UGA)^ (Fig. 6).

Taken together, our analysis highlights an evolutionary dynamic in which the mutational divergence of mtRNA^Ser(UGA)^ and mtRNA^Ser(GCU)^ along distinct evolutionary trajectories resulted in a radical rewiring of the intermolecular recognition rules underlying mitochondrial genetic code expression. Our data show how the need to maintain an indispensable molecular function under conditions of high mutation pressure can drive biological innovation even in the most conserved cellular processes, with multiple evolutionary pathways leading to functionally equivalent outcomes. Intriguingly, a similar evolutionary pattern was recently reported by Melnikov and colleagues for microsporidian parasite ribosomes, in which the erosive reduction in rRNA content was a prerequisite for the subsequent evolution of structural and functional innovations^55^. The parallels in the evolutionary trajectories shaping the ribosomes of obligate intracellular parasites and the aminoacylation system of animal mitochondria show that our preceding analysis has general relevance for our understanding of evolutionary cell biology and can help to identify and understand recurrent evolutionary patterns in organisms and molecular machineries that are exposed to mutational erosion and genome decay.

## Methods

### Cloning, expression, and purification of human mSerRS constructs

The coding sequence for human mSerRS (aa 31-518) was cloned into BamHI/XhoI sites of pGEX-6P1 vector, resulting in an N-terminally GST-tagged protein (primers used for PCR amplification and cloning are summarized in Supplementary Table 4). *E. coli* BL21(DE3) cells containing the mSerRS plasmid were grown in LB medium at 37 °C to an OD of 0.7, followed by the induction of mSerRS expression by addition of IPTG at a final concentration of 0.5 mM. Cells were harvested after shaking 16 h at room temperature (23 °C). GST-tagged mSerRS was purified by GSH-Sepharose beads (Qiagen), followed by the cleavage of the GST-tag by PreScission protease cleavage, HiTrap Heparin (GE Healthcare), and a HiLoad 16/60 Superdex 200 column (GE Healthcare) equilibrated in 20 mM HEPES pH 7.5, 150 mM NaCl, and 1 mM DTT. Purified mSerRS was concentrated and stored at −80 °C. Mutant proteins were constructed by site-directed mutagenesis and purified using the same protocol. The quality of protein preparations was validated by SDS-PAGE analysis.

### Synthesis of 5’-O-[N-(L-seryl)sulfamoyl] adenosine (SerSA)

The seryl-adenylate analogue 5’-O-[N-(L-seryl)sulfamoyl] adenosine (SerSA) was synthesized as described previously^35,56^. Briefly, 2′,3′-O-isopropylideneadenosine was heated to reflux for 5 h with a molar excess of bis(tributyltin) oxide giving its 5′-O-tributyltin ether^57^. The mixture was then treated with an excess of sulfamoyl chloride. After column chromatography, the intermediate compound was reacted with the *N*-Hydroxysuccinimide (NHS) ester of methyl (S)-( − )−3-Boc-2,2-dimethyl-4-oxazolidinecarboxylate in the presence of 1,8-Diazabicyclo(5.4.0)undec-7-ene (DBU), followed by global acidic deprotection in aqueous TFA, yielding SerSA. The final product was purified by HPLC.

### In vitro transcription of tRNAs

Genes encoding mtRNA^Ser(UGA)^ variants were cloned into the pUC-19 vector with the tRNA-coding region under the control of a T7 RNA polymerase promoter. Mutant genes were generated by site-directed mutagenesis following the QuikChange protocol (Stratagene). DNA-templates for in vitro transcription were amplified by PCR using forward and reverse primers complimentary to the T7 promoter and the 3’ end of the tRNA gene, respectively. Transcription reactions were performed in 40 mM Tris-HCl pH 8.0, 25 mM NaCl, 25 mM MgCl_2_, 2 µg/mL yeast pyrophosphatase (Roche), 1 mM Spermidine, 5 mM DTT, 18 mM GMP, 4 mM each of ATP, CTP, GTP, and UTP with 75 µg/mL T7 polymerase and DNA template at 37 °C for 6 h. Reactions were stopped by phenol/chloroform extraction followed by purification of the tRNA by 12% denaturing PAGE. tRNA was eluted from the gel in buffer containing 200 mM NaOAc, 20 mM Tris/HCl, 5 mM EDTA (pH 5.3). The eluted tRNA was then annealed by first heating to 80 °C, followed by gradual cooling to 20 °C at a rate of 2°/min. At 60 °C MgCl_2_ was added to a final concentration of 7.5 mM. The tRNA was finally ethanol-precipitated, taken up in RNase-free water and stored at −80 °C.

Like most other human mtRNAs, the mtRNA^Ser(UGA)^ is inherently unstable and prone to misfolding. As it was shown previously that the acceptor stem does not contribute to tRNA selectivity in the bovine mitochondrial serylation system^23,49^, we altered the human mtRNA^Ser(UGA)^ construct used in kinetic studies to contain two consecutive G:C in first two acceptor stem base-pairs to improve the efficiency of in vitro transcription and to increase the stability of the correctly folded tRNA. Moreover, SerRSs do not recognize the anticodon of their tRNA^Ser^ substrates^49^. To improve the stability of our tRNA construct used for structural analysis, we introduced a more stable GAAA tetraloop in lieu of the mtRNA^Ser(UGA)^ anticodon. Both alterations increased the overall yield of chargeable mtRNA^Ser(UGA)^ without reducing charging rates, suggesting that they promoted correct folding (Supplementary Fig. 1).

### Active site titration assay

The concentration of active sites was determined at RT (25 °C) in 40 μl reactions containing two different concentrations (5 and 10 μM) of human mSerRS, 20 mM L-serine, 22 nM [γ-^32^P]-ATP, in assay buffer (100 mM HEPES pH 7.5, 20 mM KCl, 10 mM MgCl_2_, 2 mM DTT, and 2 mg/mL yeast pyrophosphatase (Roche)). Reactions were initiated by adding enzyme to the assay solution in 96-well low-profile PCR plates. At different time points 5 μl reaction mix were quenched into PVDF MultiScreen filter plates (0.45 μm pore size hydrophobic, low-protein-binding membrane; Merck Millipore) containing 20 μl of 7% HClO_4_ and 80 μl of 10% charcoal slurry. Following the last time point, the slurry was mixed by pipetting and centrifuged into a 96-well flexible PET microplate (PerkinElmer) containing 150 μL of Supermix scintillation mixture (PerkinElmer). The plate was counted on a 1450 MicroBeta Micoplate Scintillation and Luminescence Counter (PerkinElmer). Kinetic data were analyzed using GraphPad Prism 8 (GraphPad Software, Inc.).

### In vitro aminoacylation

Aminoacylation reactions were carried out in an assay solution containing 50 mM HEPES pH 7.5, 60 mM KCl, 10 mM MgCl_2_, 4 mM ATP, 5 mM DTT, 4 μg/mL yeast pyrophosphatase (Roche), 1 mM Spermine, 10 μM cold L-serine, and 5 μM [^3^H]-serine (1 mCi/mL). Varying amounts of tRNA were initially mixed with assay solution, and the reaction was initiated by addition of human mSerRS (0.5 µM). At varying time intervals, 5 μL aliquots were removed and applied to a MultiScreen 96-well filter plate (0.45 μm pore size hydrophobic, low-protein-binding membrane; Merck Millipore), pre-wetted with quench solution (0.5 mg/mL salmon sperm DNA, 0.1 M EDTA, 0.3 M NaOAc (pH 3.0)). After all time points were collected, 100 μL of 20% (w/v) trichloroacetic acid (TCA) was added to precipitate nucleic acids. The plate was then washed four times with 200 μL of 5% TCA containing 100 mM cold serine, followed once by 200 μL of 95% ethanol. The plate was then dried, followed by deacylation of bound tRNAs by addition of 70 μL of 100 mM NaOH. After 10 min incubation at RT, the NaOH-solution was centrifuged into a 96-well flexible PET microplate (PerkinElmer) with 150 μL of Supermix scintillation mixture (PerkinElmer). After mixing, the radioactivity in each well of the plate was measured in a 1450 MicroBeta Micoplate Scintillation and Luminescence Counter (PerkinElmer).

### Mass photometry

Mass photometry^58^ experiments were performed in buffer composed of 20 mM HEPES pH 7.0, 100 mM NaCl, 7.5 mM MgCl_2_, and 1 mM DTT. Data were acquired at final concentrations of 25 nM mSerRS, 125 nM mtRNA^Ser(UGA)^, and 250 nM SerSA using a Refeyn One™ mass photometry system (Refeyn Ltd, Oxford, UK). The resulting video data were analyzed using DiscoverMP 2.3.0 software (Refeyn Ltd, Oxford, UK). Raw contrast values were converted to molecular mass using a standard mass calibration, and binding events combined in 2.5 kDa bin width. Binding events below 40 kDa were indistinguishable from background. Detection settings were adjusted according to the specific visualization requirements and with a background reading of buffer alone.

### tRNA sequence analysis

Sequences for genes encoding mitochondrial, prokaryotic or eukaryote cytoplasmic tRNAs were retrieved from tRNAdb/mitotRNAdb (http://trna.bioinf.uni-leipzig.de/) and the genomic tRNA database (GtRNAdb; http://gtrnadb.ucsc.edu/). Sequence alignments of tRNA genes were performed using the ClustalW function of the Molecular Evolutionary Genetics Analysis (MEGA 7.0) software^59^. Misaligned regions were curated manually based on structural characteristics of the tRNAs.

### Complex reconstitution for cryo-EM analysis

Human mSerRS and tRNA were purified individually as described above. Prior to the addition of tRNA, mSerRS was incubated with a 10-fold molar excess of SerSA ligand for 15 min at room temperature. The mSerRS-SerSA complex was then mixed with mtRNA^Ser(UGA)^ at a protein:tRNA molar ratio of 1:1.5 (corresponding to a 3-fold molar excess of tRNA over mSerRS dimer) and incubated at room temperature for 20 min. The complex was loaded onto a Superdex 200 Increase 10/300 GL column (GE Healthcare) equilibrated in 20 mM HEPES pH 7.0, 100 mM NaCl, 7.5 mM MgCl_2_, and 1 mM DTT. The mSerRS-SerSA-tRNA complex eluted in a single peak. The peak fraction containing the highest concentration of the complexes was immediately used for cryo-EM sample preparation.

### Cryo-EM sample preparation

The human mSerRS-mtRNA^Ser(UGA)^ complex was diluted to a concentration of 0.75 mg/ml and samples were mixed with 0.05% v/v Lauryl Maltose Neopentyl Glycol (Anatrace) immediately prior to plunge freezing. UltrAuFoil R1.2/1.3 300-mesh grids (Quantifoil) were plasma cleaned in a Solarus plasma cleaner (Gatan, Inc.) with a 75% nitrogen, 25% oxygen atmosphere at 15 W for 7 s. Cryo-EM grids were prepared by application of 4 μL protein sample at 4 °C in 95% humidity. The grids were manually blotted for 4-5 s using Whatman No. 1 filter paper, followed by plunge freezing in liquid ethane.

### Cryo-EM data acquisition

Cryo-EM data was collected on a Talos Arctica TEM (Thermo Fisher) operating at 200 kV in counting mode equipped with a K2 Summit direct electron detector (Gatan, Inc.). Data collection was automated using the Leginon (version 3.3) data collection software^60^. Movies were collected at a nominal magnification of 36,000x with a physical pixel size of 1.15 Å pixel^−1^. A total number of 3448 movies were collected, consisting of 200 ms frames and a total exposure time of ~11.8 s, and resulting in a cumulative exposure of 66 electrons/Å^2^. Movies were acquired using a nominal defocus range of 0.8–1.2 µm. To improve the Euler distribution of the mt-SerRS-tRNA^Ser(UGA)^ complex, about one-third of the micrographs were collected at an alpha tilt of 30 degrees. Preprocessing was performed in real-time using Warp (v1.0.7)^61^ in order to monitor data quality. Particle stacks from Warp were input to cryoSPARC (v2.13)^62^ for 2D classification (50 classes, 65% inner radius window) to assess variety of collected views.

### Human mSerRS-mtRNA^Ser(UGA)^ cryo-EM data processing

Movies were aligned and dose-weighted using MotionCor2^63^ in the Appion (v3.3)^64^ pipeline on 5 × 5 patches with an applied B-factor of 100. The aligned and dose-weighted micrographs were imported into RELION (v.3.1)^65^. The CTF was estimated from unweighted aligned images using Gctf (v1.06)^66^. Four 2D classes, representing a variety of views, were selected from prior 2D classification in cryoSPARC^62^ using a subset of the micrographs. These classes were imported into RELION and used for template picking on all micrographs and the resulting 6,890,161 picks were extracted binned 4 × 4 (4.6 Å pixel^−1^, 48-pixel box size). The 3,172,477 particle picks with an auto-picking figure of merit of >2.5 were selected and subjected to reference-free 2D classification into 200 classes using default parameters in RELION. Particle picks belonging to class averages that did not contain structural features were eliminated, resulting in a particle stack of 2.7 million particles. An initial model was obtained from a CryoSPARC ab initio reconstruction using a subset of the data (containing 784,419 particles) and used as input for 3D auto-refinement of the particles in RELION. Subsequently, the refined stack was input to 3D classification (4 classes, tau-value of 4, 25 iterations, without alignment) and a single high-resolution class, containing 655,319 particles, was selected for further processing. The particles were re-centered and re-extracted at a binning of 2 × 2 (2.3 Å pixel^−1^, 96-pixel box size), input to 3D auto-refinement and a subsequent 3D classification step (4 classes, tau-value of 4, 25 iterations, without alignment). One high-resolution class was selected, which contained 200,065 particles. The particles were re-centered and re-extracted at full resolution (1.15 Å pixel^−1^, 192-pixel box size) and auto-refined to a nominal resolution of 4.2 Å. The stack was grouped by beam shift and iteratively CTF and 3D auto-refined to convergence, yielding a final reconstruction at a reported nominal resolution of 3.6 Å. Subsequently, the local resolution was estimated using RELION and the 3D Fourier Shell Correlation was calculated using the 3D FSC server (https://3dfsc.salk.edu)^67^.

### Atomic modeling and refinement of the cryo-EM structures

The crystal structure of mSerRS-SerSA complex (PDB 7TZB) and the *E. coli* tRNA^Phe^ structure (PDB 3L0U)^68^ were used as starting models and were rigid body fit into the EM density maps. The model for mtRNA^Ser(UGA)^ was trimmed to appropriate chain lengths and rebuilt in regions that deviated substantially from the *E. coli* tRNA^Phe^ structure. The geometry of the tRNA model was optimized using ERRASER^69^ through the Rosetta Online Server (https://rosie.graylab.jhu.edu/). The models were real-space refined in Coot (v0.9)^70^, restrained to ideal geometry, secondary structure and Geman-McClure distance restraints generated in ProSMART (v0.8)^71^ from the input models. The models were iteratively real-space refined in Coot^70^ and in Phenix (v1.20.1)^72^ (by rigid body and global minimization) using Ramachandran and secondary structural restraints. The model was further optimized for compliance to geometric constraints using MolProbity^73^ as guidance and by geometry minimization in Phenix (v1.20.1)^72^. MolProbity was used to assess the quality of the final model and report validation statistics in Supplementary Table 1.

### Figure generation

Figures were rendered in ChimeraX^74^.

### Quantification and statistical analysis

The statistical analysis of the cryo-EM data processing, model building, and model refinement is described in Method details and summarized in Supplementary Table 1.

### Reporting summary

Further information on research design is available in the Nature Portfolio Reporting Summary linked to this article.

### Supplementary information

Supplementary Information

Reporting Summary

Peer Review

### Source data

Source Data

## Data Availability

The data supporting the findings of this study are available from the corresponding authors upon reasonable request. The cryo-EM map of mSerRS-mtRNA^Ser(UGA)^ has been deposited in the Electron Microscopy Data Bank (EMDB) under the accession code EMD-29070. Atomic coordinates of the model have been deposited in the Protein Data Bank (PDB) under accession code 8FFY. The atomic coordinates used for molecular replacement or structural comparison were downloaded from the PDB: 1SER, 3L0U, 4TRA, 5UD5, 6YDP, 6ZM6, 7ONU, 7TZB, 7U2A, 7U2B. All tRNA gene sequences were retrieved from the tRNAdb/mitotRNAdb (http://trna.bioinf.uni-leipzig.de/), genomic tRNA database (GtRNAdb; http://gtrnadb.ucsc.edu/) or the National Center for Biotechnology Information database (NCBI; NC027264). Source data for the figures and supplementary figures are provided as a Source Data file. Source data are provided with this paper.
