# Supplementary material for: Structural basis for a degenerate tRNA identity code and the evolution of bimodal specificity in human mitochondrial tRNA recognition
Source: Nat Commun. 2023 Aug 9;14:4794. doi: 10.1038/s41467-023-40354-2 (PMC10412605; doi:10.1038/s41467-023-40354-2)
Supplement: Supplementary file 1 — Supplementary Information [file 41467_2023_40354_MOESM1_ESM.pdf]

SUPPLEMENTARY INFORMATION

Structural basis for a degenerate tRNA identity code and the evolution of bimodal specificity in human mitochondrial tRNA recognition

Bernhard Kuhle, Marscha Hirschi, Lili K. Doerfel, Gabriel C. Lander, Paul Schimmel

This file includes:

Supplementary Figures 1–9

Supplementary Tables 1-4

Supplementary References

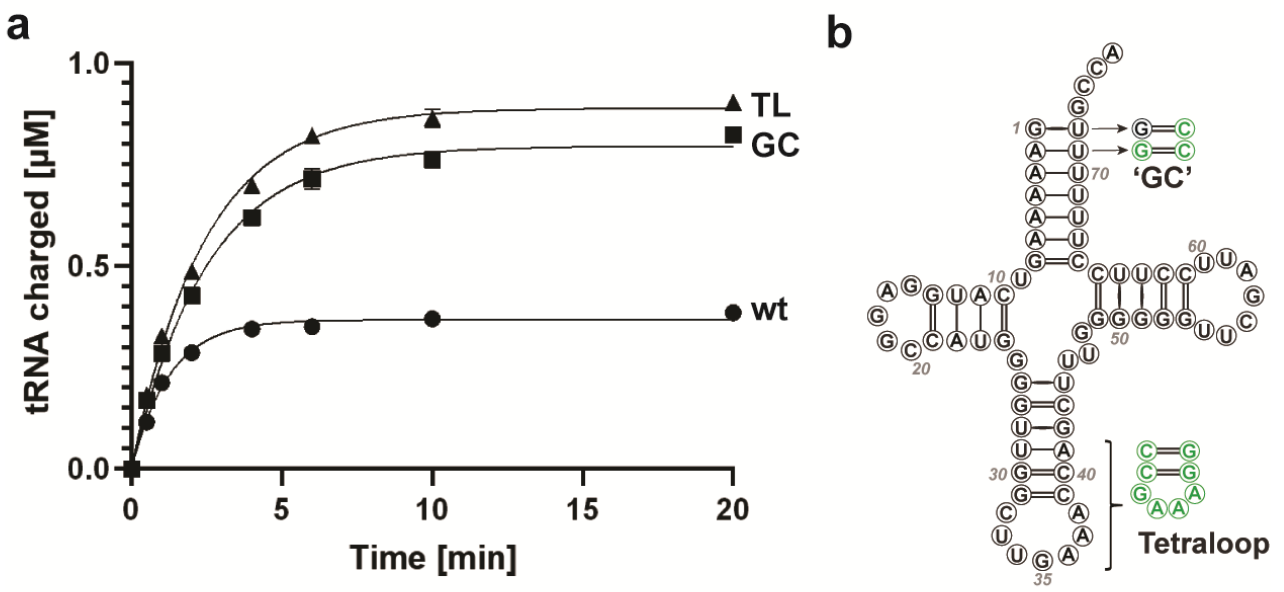

Supplementary Figure 1. Human mtRNA$^{Ser(UGA)}$ variants used for structure and kinetic analyses. Related to Fig. 1. Charging activities (**a**) and secondary structures (**b**) for variants of human mtRNA$^{Ser(UGA)}$ used in this study. Aminoacylation reactions contained 0.25 µM mSerRS and 2 µM tRNA and were carried out at room temperature. Data points represent the mean, and error bars represent the SEM from three independent experiments.

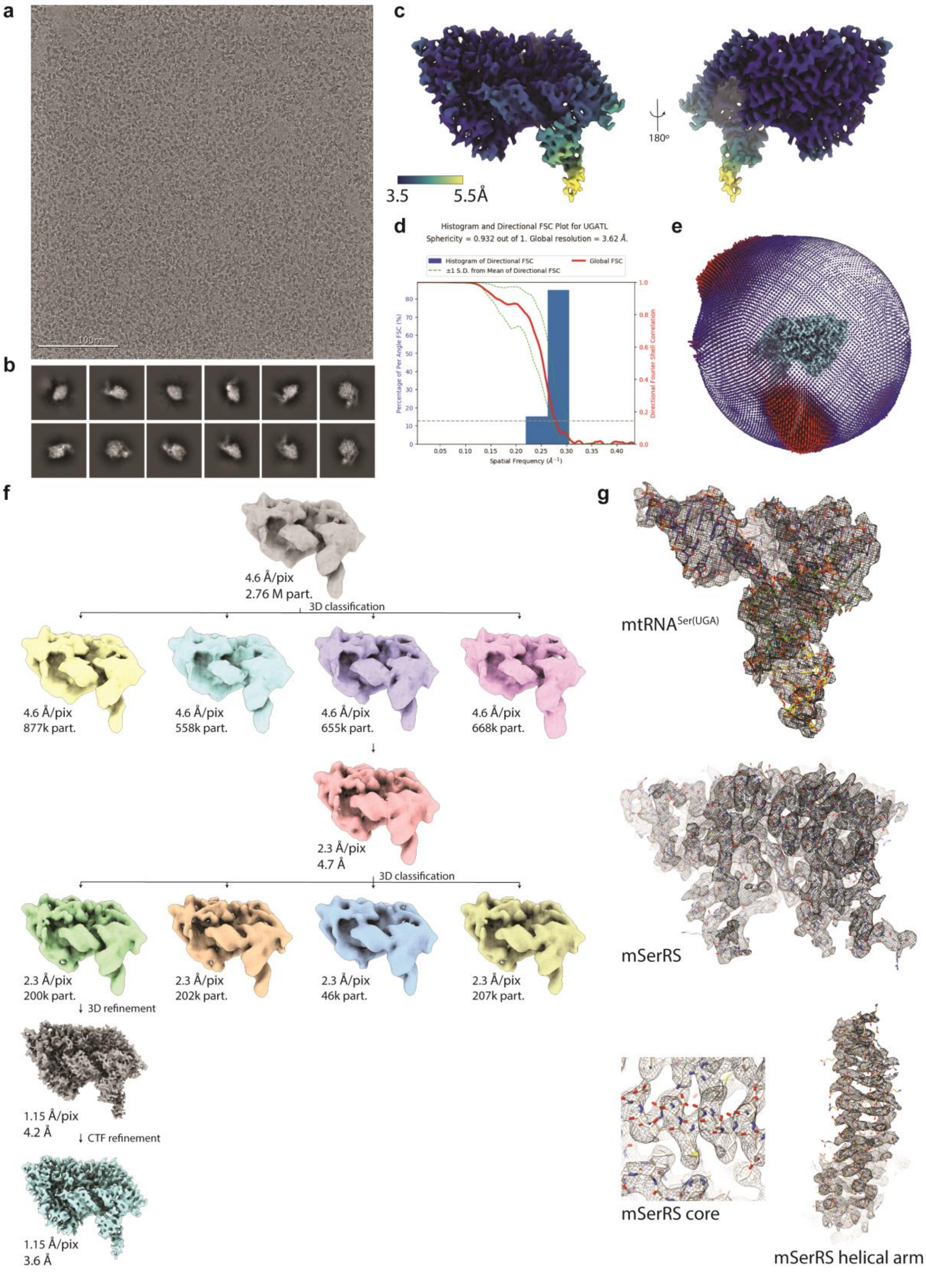

Supplementary Figure 2. Cryo-EM data collection and processing of human mSerRS-mtRNA$^{Ser(UGA)}$. Related to Fig. 1. **a**. Representative micrograph from a total of 3448 micrographs taken. **b.** Selected 2D class averages showing secondary structural information. **c.** Local resolution of the mSerRS-mtRNA$^{Ser(UGA)}$ reconstruction. **d.** Fourier Shell Correlation between half maps. **e.** Euler distribution of the final reconstruction. **f.** Data processing scheme for mSerRS-mtRNA$^{Ser(UGA)}$. **g.** Components of the mSerRS-mtRNA$^{Ser(UGA)}$ complex fit into the EM density.

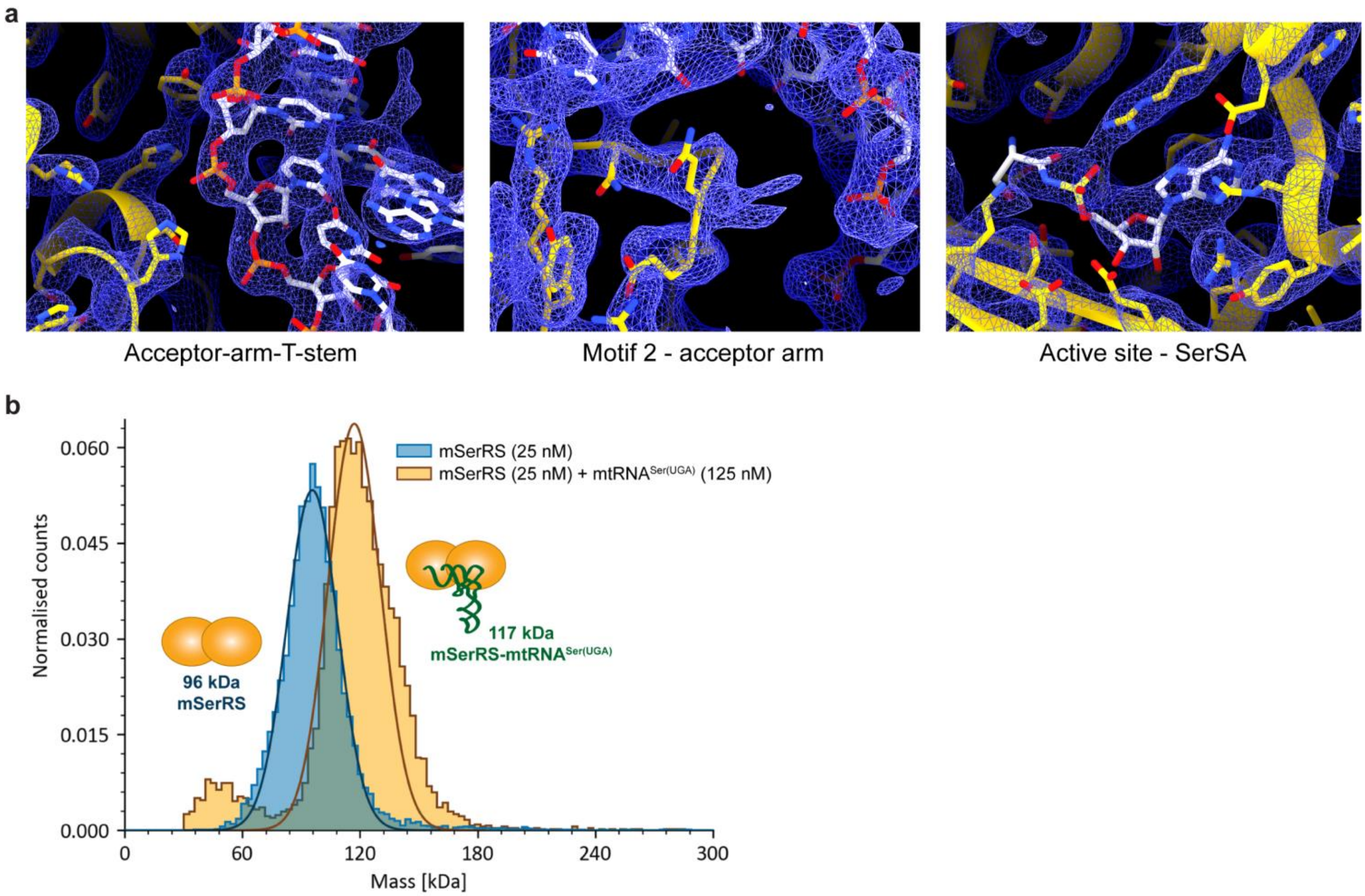

Supplementary Figure 3. Related to Fig. 1. **a.** Representative EM densities of the mSerRS-tRNA$^{Ser(UGA)}$ complex. Close-up views of the EM densities for the mSerRS-mtRNA$^{Ser(UGA)}$ complex shown with the underlying refined atomic models. The protein is colored in yellow, tRNA in light gray. **b.** Mass photometry results for mSerRS-mtRNA$^{Ser(UGA)}$ complex formation. The mSerRS dimer has a calculated molecular mass of 108 kDa, the calculated molecular mass of mtRNA$^{Ser(UGA)}$ is 23.7 kDa.

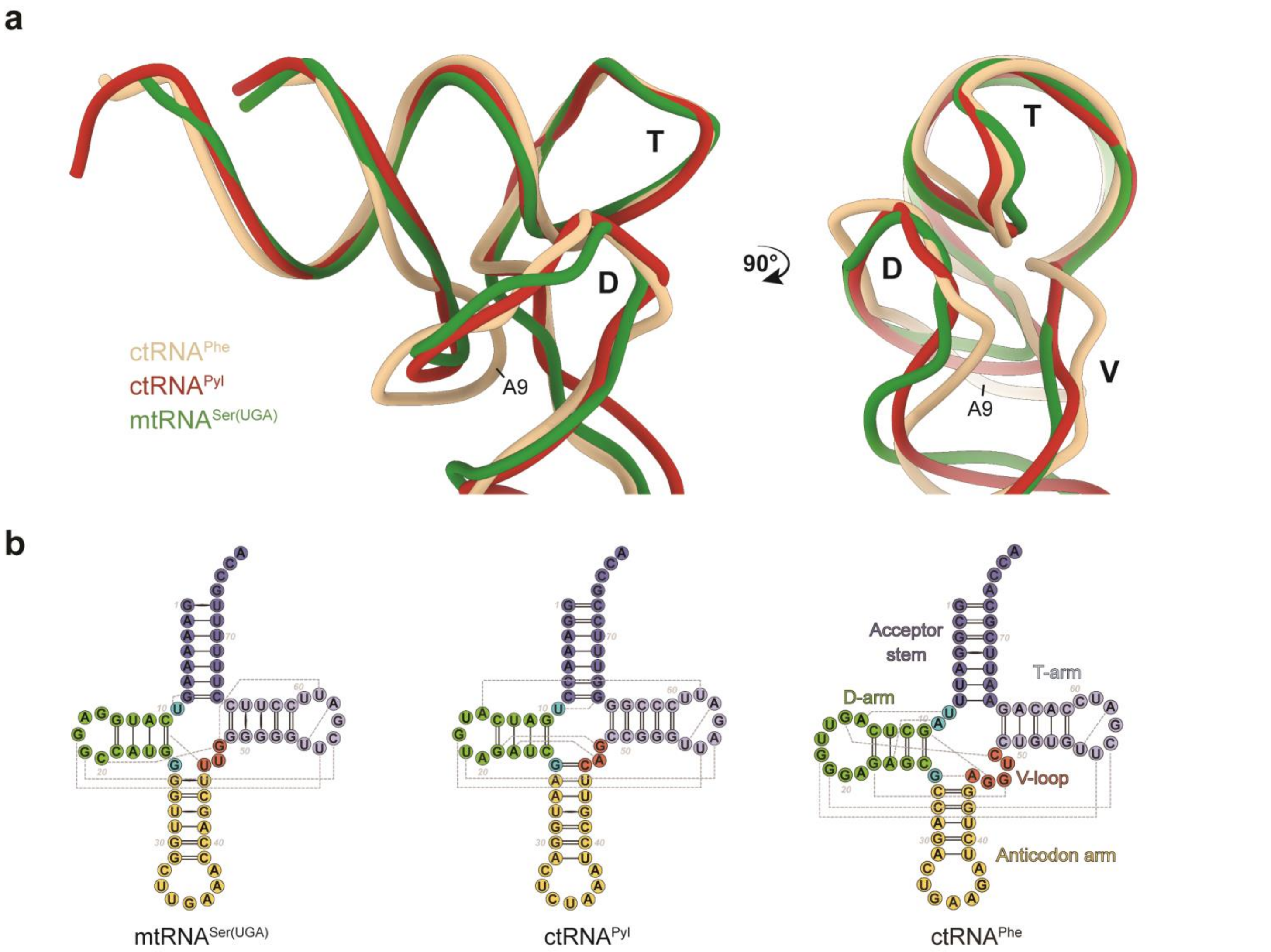

Supplementary Figure 4. Convergent structural features in human mtRNA$^{Ser(UGA)}$ and prokaryote tRNA^{Pyl}. Related to Fig. 2. **a**. Structural overlay between the human mtRNA$^{Ser(UGA)}$ (green), *M. mazei* tRNA^{Pyl} (red) (PDB 5UD5)[1], and *S. cerevisiae* tRNA^{Phe} (beige) (PDB 4TRA)[2]. A9, which forms part of the two-nucleotide linker between acceptor-stem and D-stem in canonical tRNAs is missing in mtRNA$^{Ser(UGA)}$ and tRNA^{Pyl}, shifting the entire D-arm upward and allowing their reduced D-loops to form canonical tertiary interactions with the T-loop. **b**. Secondary structure comparison between human mtRNA$^{Ser(UGA)}$ (left), *M. mazei* tRNA^{Pyl} (middle), and *S. cerevisiae* tRNA^{Phe} (right). Tertiary interactions are indicated by dashed lines.

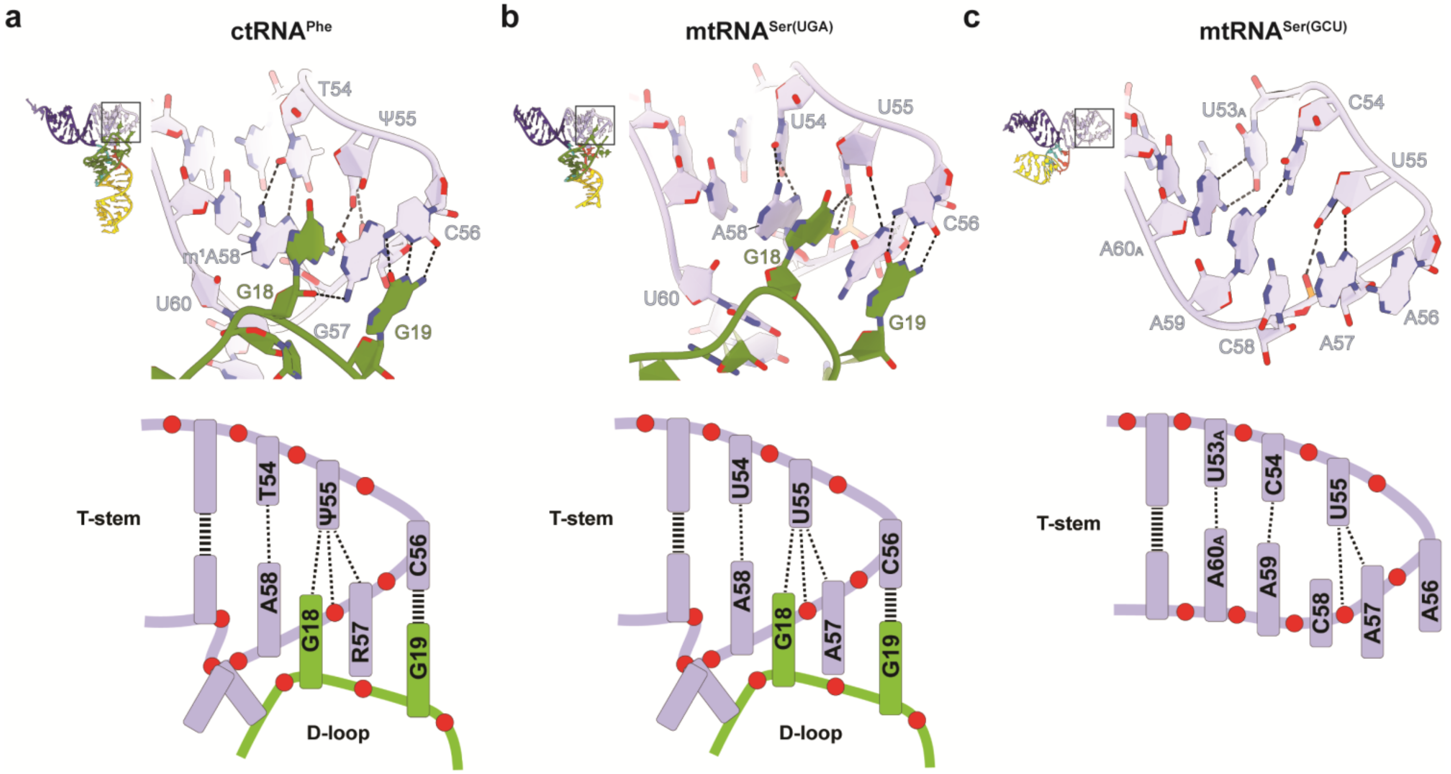

Supplementary Figure 5. Elbow structure comparison between tRNA^{Phe}, human mtRNA$^{Ser(UGA)}$, and human mtRNA$^{Ser(GCU)}$. Related to Fig. 2. Atomic structural (top) and schematic (bottom) presentation of the T-loop-D-loop ('elbow') region in the canonical cytoplasmic tRNA^{Phe} from *S. cerevisiae* (PDB 4TRA)[2] (a), human mtRNA$^{Ser(UGA)}$ (b) and human mtRNA$^{Ser(GCU)}$ (PDB 7U2B)[3] (c). The T-loop is colored in light blue, the D-loop is colored in green. In mature human mtRNA$^{Ser(UGA)}$, the T-loop contains canonical tRNA modifications in positions U54 (m^5U), U55 (ψ), and A58 (m^1A)[4]. The T-loop of mature human mtRNA$^{Ser(GCU)}$ is unmodified[4].

a

```
mtdbD00000544|H.sapiens|Ser1|GCT   GAGAAAGCT................CACAAGAACTGCTAACTCATGCCC.CCATGTCTAACAA.CATGGCTTTCTCA
mtdbD00000542|H.sapiens|Arg|TCG    TGGTATATAGTTTAAA.CAA....AACGAATGATTTCGACTCATTAAAT.TATGATAA......TCATATTTACCAA
mtdbD00000537|H.sapiens|Tyr|GTA    GGTAAAATGGCTGAG......TGAAGCATTGGACTGTAAATCTAAAGAC.AGGGGTTAG.....GCCTCTTTTTACC
mtdbD00000536|H.sapiens|Cys|GCA    AGCTCCGAGGTGATTT.......TCATATTGAATTGCAAATTCGAAGAA.GCAGCTTC.AAA..CCTGCCGGGGCTT
mtdbD00000547|H.sapiens|Thr|TGT    GTCCTTGTAGTATAA...AC.TAATACACCAGTCTTGTAAACCGGAGAT.GAAAACCT......TTTTCCAAGGACA
mtdbD00000541|H.sapiens|Gly|TCC    ACTCTTTTAGTATAAA.TAG....TACCGTTAACTTCCAATTAACTAGT.TTTGACAACA.T..TCAAAAAAGAGTA
mtdbD00000539|H.sapiens|Asp|GTC    AAGGTATTAGAAAAAC.CAT....TTCATAACTTTGTCAAAGTTAAATT.ATAGGCTA.AAT..CCTATATATCTTA
mtdbD00000532|H.sapiens|Met|CAT    AGTAAGGTCAGCTAAA.....TAAGCTATCGGGCCCATACCCCGAAAAT.GTTGGTATA....CCCTTCCCGTACTA
mtdbD00000548|H.sapiens|Pro|TGG    CAGAGAATAGTTTAAA.TTA...GAATCTTAGCTTTGGGTGCTAATGGT.GGAGTTAAAG....ACTTTTTCTCTGA
mtdbD00000533|H.sapiens|Trp|TCA    AGAAATTTAGGTTAA...ATACAGACCAAGAGCCTTCAAAGCCCTCAGT.AAGTTGCAA.....TACTTAATTTCTG
mtdbD00000534|H.sapiens|Ala|TGC    AAGGGCTTAGCTTAAT.TAA....AGTGGCTGATTTGCGTTCAGTTGAT.GCAGAGTGGGGT..TTTGCAGTCCTTA
mtdbD00000546|H.sapiens|Glu|TTC    GTTCTTGTAGTTGAAA.TAC....AACGATGGTTTTTCATATCATTGGT.CGTGGTTGTAGT..CCGTGCGAGAATA
mtdbD00000543|H.sapiens|His|GTG    GTAAATATAGTTTAAC.CAA....AACATCAGATTGTGAATCTGACAAC.AGAGGCTTACGA..CCCCTTATTTACC
mtdbD00000530|H.sapiens|Ile|GAT    AGAAATATGTCTGATA.AA.....AGAGTTACTTTGATAGAGTAAATAATAGGAGCTTAAAC..CCCCTTATTTCTA
mtdbD00000528|H.sapiens|Val|TAC    CAGAGTGTAGCTTAACACAA....AGCACCCAACTTACACTTAGGAGAT.TTCAACTTAA...CTTGACCGCTCTGA
mtdbD00000538|H.sapiens|Ser2|TGA   GAAAAAGT.CATGGA...GG.CCATGGGGTTGGCTTGAAACCAGCTTTG.GGGGGTTCGATT..CCTTCCTTTTTTG
mtdbD00000540|H.sapiens|Lys|TTT    CACTGTAAAGCTAACT.......TAGCATTAACCTTTTAAGTTAAAGATTAAGAGAACCAACACCTCTTTACAGTGA
mtdbD00000545|H.sapiens|Leu1|TAG   ACTTTTAAAGGATAAC.AGC..TATCCATTGGTCTTAGGCCCCAAAAAT.TTTGGTGCAACT..CCAAATAAAAGTA
mtdbD00000527|H.sapiens|Phe|GAA    GTTTATGTAGCTTACC.TCCTCAAAGCAATACACTGAAAATGTTTAGAC.GGGCTCACAT....CACCCCATAAACA
mtdbD00000531|H.sapiens|Gln|TTG    TAGGATGGGGTGTGAT.AGG.TGGCACGGAGAATTTTGGATTCTCAGGG.ATGGGTTCGATT..CTCATAGTCCTAG
mtdbD00000535|H.sapiens|Asn|GTT    TAGATTGAAGCCAGTT.GAT.TAGGGTGCTTAGCTGTTAACTAAGTGTTTGTGGGTTTAAGT..CCCATTGGTCTAG
mtdbD00000529|H.sapiens|Leu2|TAA   GTTAAGATGGCAGAGCCCGGTAATCGCATAAAACTTAAAACTTTACAGTCAGAGGTTCAATT..CCTCTTCTTAACA
tdbD00000785|S.cerevisiae|Phe|GAA  GCGGATTTAGCTCAGT.TGG.GAGAGCGCCAGACTGAAGATCTGGAGGTCCTGTGTTCGATC..CACAGAATTCGCA
                                   3'-AS         D-loop          Anticodon-loop   V-loop     T-loop      5'-AS
```

b

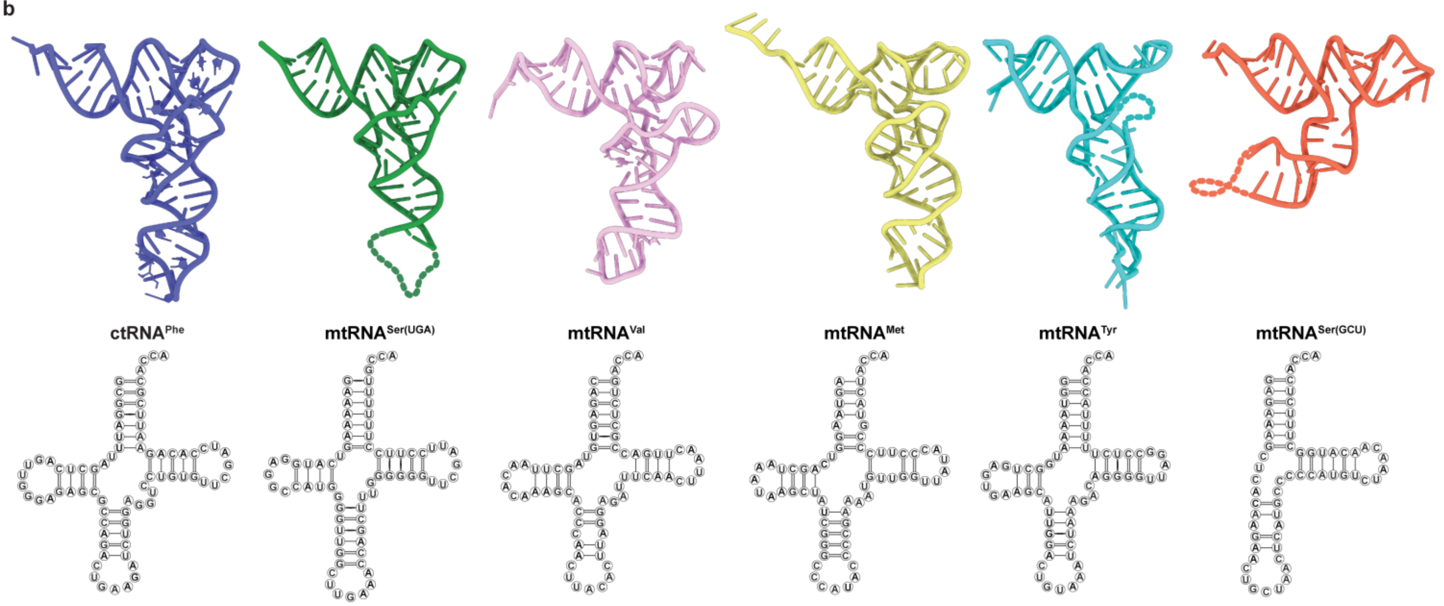

Supplementary Figure 6. mtRNA$^{Ser(UGA)}$ and mtRNA$^{Ser(GCU)}$ lie on opposite ends of the structural spectrum of human mtRNAs. Related to Fig. 3. **a.** Structure-based sequence alignment of the 22 human mitochondrial tRNAs and *S. cerevisiae* cytoplasmic tRNA^{Phe}. The 22 mtRNAs are ordered according to their 'length distance' from the canonical 73 nucleotides (excluding the 3'-CCA) of tRNA^{Phe} from the shortest mtRNA at the top to the longest at the bottom. Human mtRNA$^{Leu(UAA)}$ (75 nt) is the only human mtRNA that has increased in length relative to the canonical tRNA^{Phe}. **b**. Tertiary (top) and secondary (bottom) structure comparison between *S. cerevisiae* cytoplasmic tRNA^{Phe} (blue; PDB 4TRA)[2], and human mtRNA$^{Ser(UGA)}$ (green), mtRNA^{Val} (pink; PDB 6ZM6)[5], mtRNA^{Met} (yellow; PDB 6YDP)[6], mtRNA^{Tyr} (cyan; PDB 7ONU)[7], and mtRNA$^{Ser(GCU)}$ (tomato; PDB 7U2B)[3]. The tRNAs are ordered according to their relative position in the alignments shown in a. Among these mtRNAs, mtRNA$^{Ser(UGA)}$ is the only one capable of forming the G19:C56 tertiary pair which defines the canonical elbow structure, while mtRNA$^{Ser(GCU)}$ is the only one completely lacking the D-arm and a tertiary core. It should be noted that all mtRNAs shown here are part of complexes with other RNAs and/or protein partners (not shown), which may stabilize specific structural states.

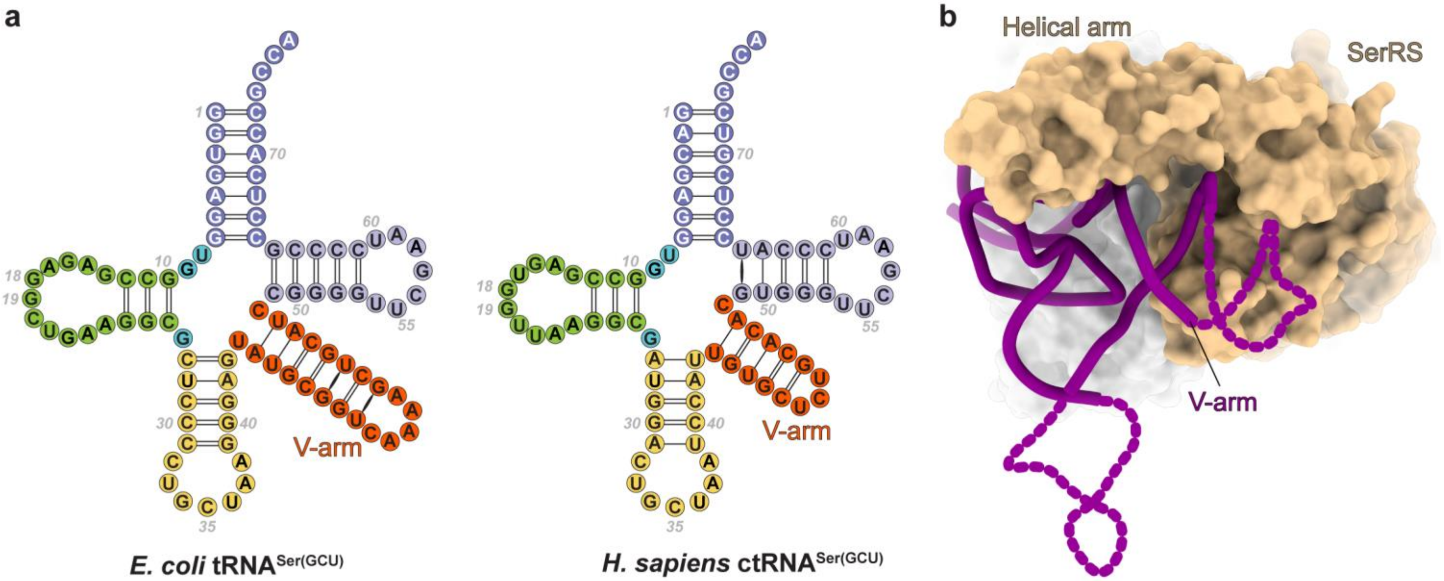

Supplementary Figure 7. The extended V-arm serves as the primary identity element in canonical SerRS-tRNA^{Ser} interactions. a. Secondary structures of canonical tRNA$^{Ser(GCU)}$ from *E. coli* (left) and the human cytoplasm (right). The extended V-arm in both tRNAs is shown in orange. **b.** Structure of the *T. thermophilus* SerRS-tRNA^{Ser} complex (PDB 1SER). SerRS is shown in surface presentation. The tRNA is shown in cartoon presentation and colored purple. Missing regions of the V-arm and anticodon arm are indicated by purple dashed lines.

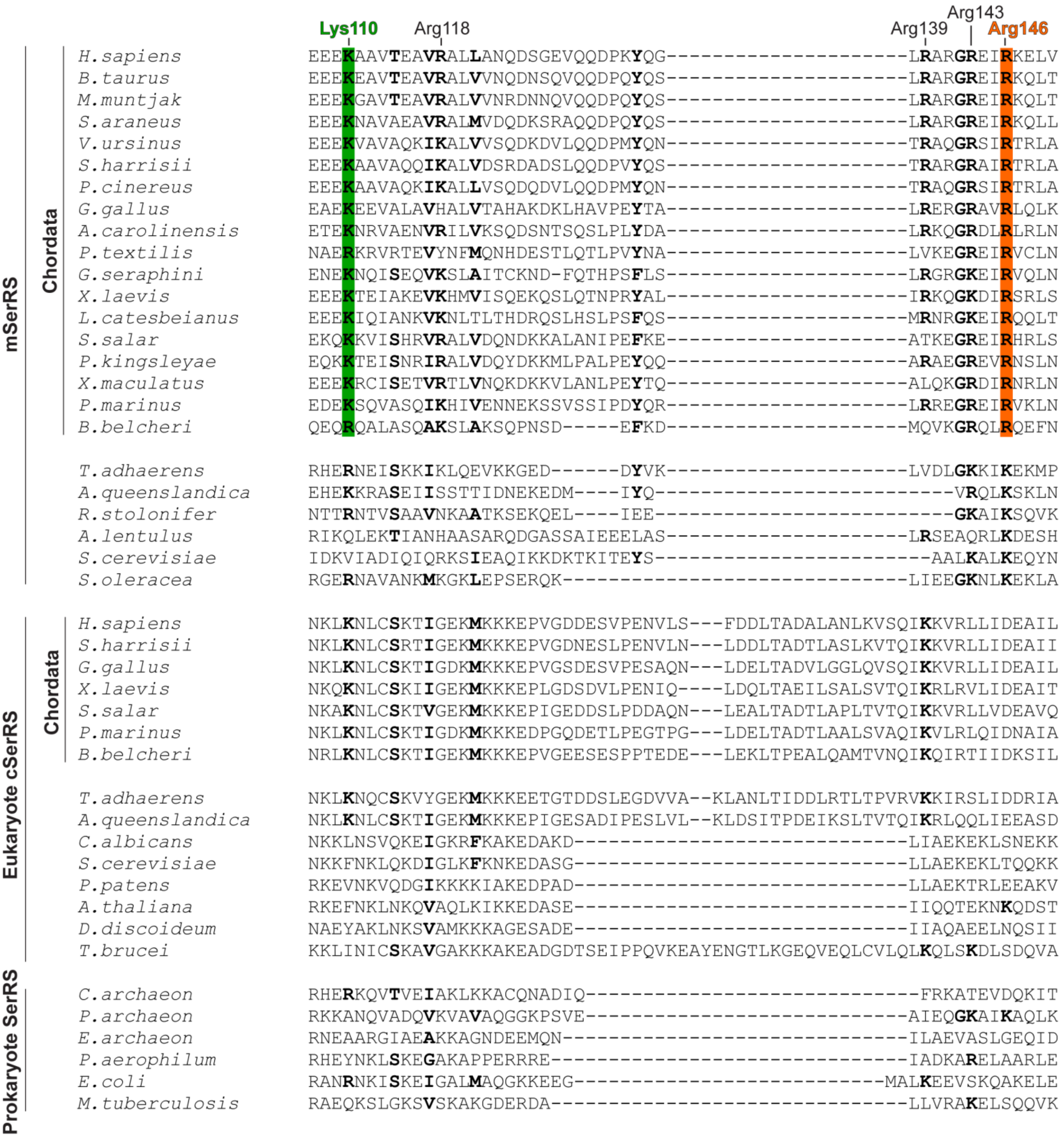

Supplementary Figure 8. Conservation of the mtRNA^{Ser} elbow-binding interface in mSerRS. Related to Fig. 4 and 5. **a.** Multiple sequence alignment of the helical arm from prokaryotic, eukaryote cytoplasmic (c), and mitochondrial (m) SerRSs. Residues forming the helical arm binding pocket in human mSerRS are indicated in bold. The catalytically important Lys110 and Arg146 are highlighted in green and tomato, respectively.

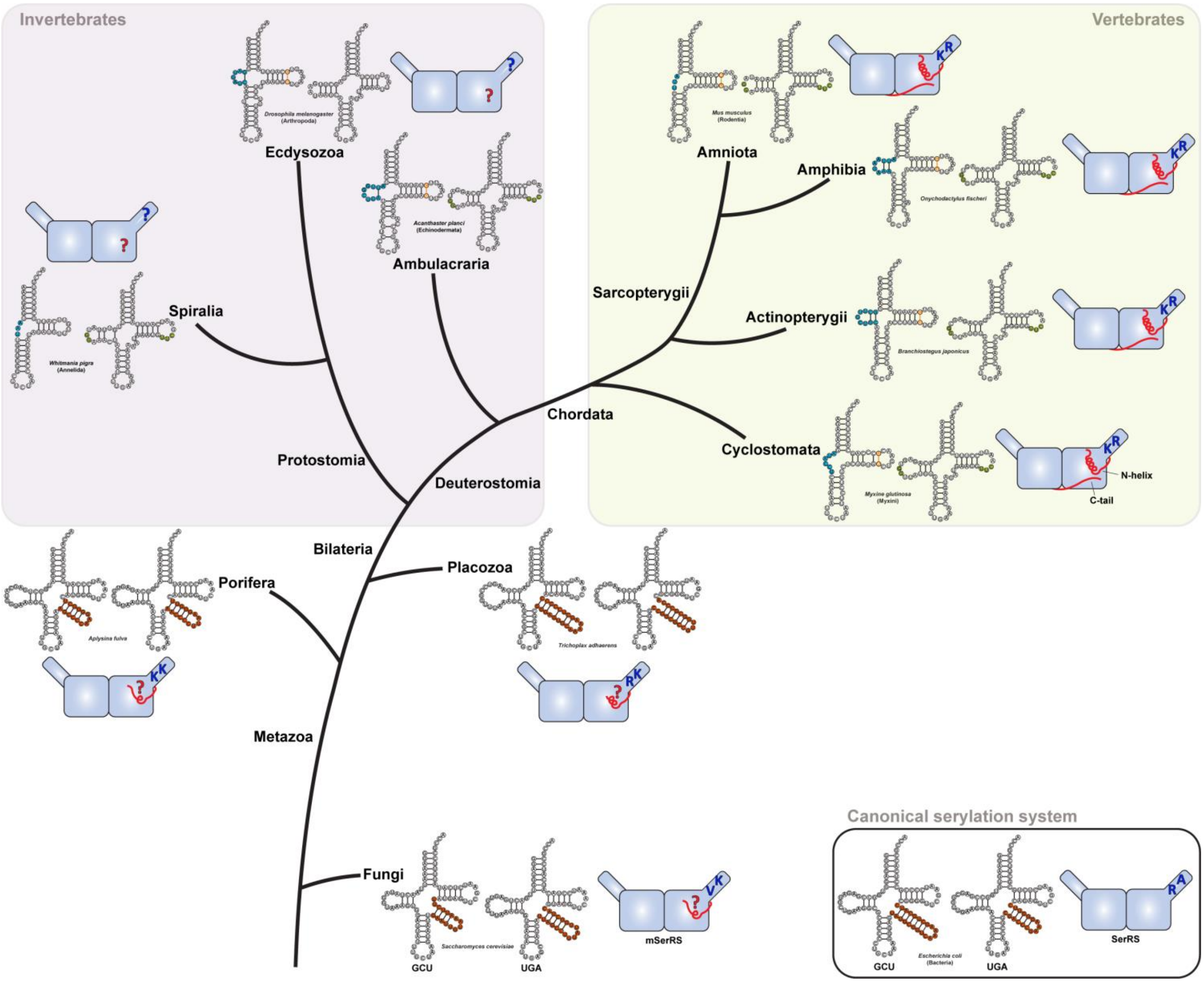

Supplementary Figure 9. Schematic representation of the phylogenetic distribution mSerRS and mtRNA^{Ser} characteristics and recognition elements in animal mitochondria. Related to Fig. 5. Characteristic features and recognition elements of mtRNA$^{Ser(UGA)}$ and mtRNA$^{Ser(GCU)}$ and mSerRS identified in mammals (class of the clade Amniota) are conserved in extant vertebrate species. This includes the loss of the extended V-arm in both isoacceptors, the extended T-arm in a more degenerated mtRNA$^{Ser(GCU)}$, a near-canonical mtRNA$^{Ser(UGA)}$, as well as the N-helix and C-tail (red lines), Lys110 (K), and Arg146 (R) in mSerRS. By contrast, non-bilaterian animal mtRNA^{Ser} isoacceptors have retained canonical tRNA^{Ser} characteristics and their mSerRSs do not yet contain the complete set of anchor points for mtRNA^{Ser} binding. Rudiments of the N-helix may already be present as part of the mitochondrial targeting signal sequence. Interestingly, invertebrates (Protostomia and Ambulacraria) show many of the characteristics found in vertebrates, including loss of the extended V-arm and asymmetric divergence of the two mtRNA^{Ser} isoacceptors. Notably, many invertebrate species contain an essential mSerRS homologue called SLIMP[8], which forms a heterodimeric complex with mSerRS and is required for mtRNA^{Ser} aminoacylation in drosophila[9]. How invertebrate mtRNA^{Ser} are recognized by their cognate synthetase is currently unknown. The bacterial (*E. coli*) tRNA^{Ser} and SerRS are shown as representatives of the ancestral canonical serylation system in which both isoacceptors are recognized through their extended V-arm (orange).

Supplementary Table 1.
Cryo-EM data collection, refinement, and validation statistics for mSerRS-tRNA$^{Ser(UGA)}$ (related to Fig. 1).

**Data collection and processing**	
Microscope	Talos Arctica
Voltage (kV)	200
Nominal magnification	x36,000
Exposure navigation	Image shift
Electron exposure (e^{-} Å^{-2})	66
Exposure rate (e^{-} pixel^{-1} s^{-1})	5
Detector	K2 Summit
Defocus range (µm)	-0.8 to -1.2
Pixel size (Å)	1.15
Micrographs	3448
Automation software	Leginon
Total extracted particles (no.)	7,218,136
Refined particles (no.)	6,231,523
**Reconstruction**	
Final particles (no.)	881,655
Symmetry imposed	C1
Map resolution (Å)<br>FSC 0.5 (unmasked/masked)<br>FSC 0.143 (unmasked/masked)	3.6<br>4.4/3.6<br>4.0/3.6
Map resolution range (Å)	3.7-7.7
Applied B-factor (Å^2)	-100
3D FSC Sphericity (%)	0.932
**Refinement**	
Refinement package	Phenix (v.1.20.1)
Initial model used (PDB code)	mSerRS (7TZB)
Model composition<br>Protein residues<br>Nucleotide residues	 770<br>61
Map Correlation Coefficient	0.61
Average *B* factors (Å^2)	128
R.m.s. deviations<br>Bond lengths (Å)<br>Bond angles (°)	 0.002<br>0.509
Ramachandran plot<br>Favored (%)<br>Allowed (%)<br>Disallowed (%)	 99.87<br>0.13<br>0.0
Poor rotamers (%)	0.0
MolProbity score	1.18
Clashscore (all atoms)	3.95
C-beta deviations	0.0
CaBLAM outliers (%)	1.2
EMRinger score	1.56

Supplementary Table 2.

Kinetics of aminoacylation of mtRNA$^{Ser(UGA)}$ variants by mSerRS (related to Fig. 5d).

tRNA variants	k_{obs} (10^{-3} s^{-1})	k_{obs} *relative*
WT (‘GC’)	64.4 ± 2.1	1.00
G19C	19.5 ± 0.6	0.30
G19A/C56U	59.5 ± 3.6	0.92
G19C/C56G	57.0 ± 3.2	0.89
C20U	63.8 ± 1.3	0.99
U63C	49.8 ± 2.4	0.77
U64C	40.1 ± 1.2	0.62

Means and standard errors were calculated from three independent experiments. Source data are provided as a Source Data file.

Supplementary Table 3.
Kinetics of aminoacylation of mtRNA$^{Ser(UGA)}$ by mSerRS mutants (related to Fig. 5f).

mSerRS	k_{cat} (min^{-1})	K_m μM	k_{cat}/K_m (min^{-1} $μM^{-1}$)	k_{cat}/K_m relative
WT	5.49 ± 0.13	0.89 ± 0.06	6.19	1.00
K110A	1.58 ± 0.04	2.02 ± 0.16	0.78	0.13
R118A	5.38 ± 0.02	1.03 ± 0.01	5.24	0.85
R139A	4.68 ± 0.08	1.51 ± 0.05	3.11	0.50
R143A	5.50 ± 0.06	1.37 ± 0.04	4.01	0.65
3xRA	2.67 ± 0.1	2.28 ± 0.05	1.17	0.19
R146A	5.86 ± 0.07	1.19 ± 0.04	4.91	0.79

Means and standard errors were calculated from three independent experiments. Source data are provided as a Source Data file.

Supplementary Table 4.
Primers used in this study.

Human SARS2 PCR primer 5’<br>GGGGCCCCTGGGATCCACTACAGAGAAACGAAACCGGAACC
Human SARS2 PCR primer 3’<br>GATGCGGCCGCTCGAGTTAGCTTACAGCAGGCTGGCCAG
Human mtRNA$^{Ser(UGA)}$ PCR primer 5‘<br>TTGGCTTGAAACCAGCTTTGGGGGGTTCGATTCCTTCCTTTTTTGCCATAGAGGATCCCCGGGTAC
Human mtRNA$^{Ser(UGA)}$ PCR primer 3‘<br>CTGGTTTCAAGCCAACCCCATGGCCTCCATGACTTTTTCTATAGTGAGTCGTATTAATTACTGCAG
Human mtRNA$^{Ser(UGA)-TL}$ PCR primer 5‘<br>GGGTCCGAAAGGGCTTTGGGGGGTTCGATTCCTTCCTTTTTTG
Human mtRNA$^{Ser(UGA)-TL}$ PCR primer 3‘<br>AAGCCCTTTCGGACCCCATGGCCTCCATGACTTTTTCTATAG
T7-promoter template PCR primer 5‘<br>GGATCCTAATACGACTCACTATAG
Human mtRNA$^{Ser(UGA)}$ template PCR primer 3‘<br>TGGCAAAAAAGGAAGGAATCGAACCC
Human mtRNA$^{Ser(UGA)}$ (‘GC’) template PCR primer 3‘<br>TGGCGGAAAAGGAAGGAATCGAACC

Supplementary References

1 Suzuki, T. *et al.* Crystal structures reveal an elusive functional domain of pyrrolysyl-tRNA synthetase. *Nat Chem Biol* **13**, 1261-1266, doi:10.1038/nchembio.2497 (2017).

2 Westhof, E., Dumas, P. & Moras, D. Restrained refinement of two crystalline forms of yeast aspartic acid and phenylalanine transfer RNA crystals. *Acta Crystallogr A* **44 (Pt 2)**, 112-123 (1988).

3 Kuhle, B., Hirschi, M., Doerfel, L. K., Lander, G. C. & Schimmel, P. Structural basis for shape-selective recognition and aminoacylation of a D-armless human mitochondrial tRNA. *Nat Commun* **13**, 5100, doi:10.1038/s41467-022-32544-1 (2022).

4 Suzuki, T. *et al.* Complete chemical structures of human mitochondrial tRNAs. *Nat Commun* **11**, 4269, doi:10.1038/s41467-020-18068-6 (2020).

5 Itoh, Y. *et al.* Mechanism of membrane-tethered mitochondrial protein synthesis. *Science* **371**, 846-849, doi:10.1126/science.abe0763 (2021).

6 Kummer, E. & Ban, N. Structural insights into mammalian mitochondrial translation elongation catalyzed by mtEFG1. *EMBO J* **39**, e104820, doi:10.15252/embj.2020104820 (2020).

7 Bhatta, A., Dienemann, C., Cramer, P. & Hillen, H. S. Structural basis of RNA processing by human mitochondrial RNase P. *Nat Struct Mol Biol* **28**, 713-723, doi:10.1038/s41594-021-00637-y (2021).

8 Guitart, T. *et al.* New aminoacyl-tRNA synthetase-like protein in insecta with an essential mitochondrial function. *J Biol Chem* **285**, 38157-38166, doi:10.1074/jbc.M110.167486 (2010).

9 Picchioni, D. *et al.* Mitochondrial Protein Synthesis and mtDNA Levels Coordinated through an Aminoacyl-tRNA Synthetase Subunit. *Cell Rep* **27**, 40-47 e45, doi:10.1016/j.celrep.2019.03.022 (2019).
