# Supplementary material for: Structural basis for a degenerate tRNA identity code and the evolution of bimodal specificity in human mitochondrial tRNA recognition
Source: Nat Commun. 2023 Aug 9;14:4794. doi: 10.1038/s41467-023-40354-2 (PMC10412605; doi:10.1038/s41467-023-40354-2)
Supplement: Supplementary file 2 — Reporting Summary [file 41467_2023_40354_MOESM2_ESM.pdf]

nature portfolio

Corresponding author(s): Bernhard Kuhle

Last updated by author(s): Jul 11, 2023

# Reporting Summary

Nature Portfolio wishes to improve the reproducibility of the work that we publish. This form provides structure for consistency and transparency in reporting. For further information on Nature Portfolio policies, see our Editorial Policies and the Editorial Policy Checklist.

## Statistics

For all statistical analyses, confirm that the following items are present in the figure legend, table legend, main text, or Methods section.

| n/a | Confirmed | |
|---|---|---|
| ☐ | ☒ | The exact sample size (*n*) for each experimental group/condition, given as a discrete number and unit of measurement |
| ☒ | ☐ | A statement on whether measurements were taken from distinct samples or whether the same sample was measured repeatedly |
| ☒ | ☐ | The statistical test(s) used AND whether they are one- or two-sided<br>*Only common tests should be described solely by name; describe more complex techniques in the Methods section.* |
| ☒ | ☐ | A description of all covariates tested |
| ☒ | ☐ | A description of any assumptions or corrections, such as tests of normality and adjustment for multiple comparisons |
| ☐ | ☒ | A full description of the statistical parameters including central tendency (e.g. means) or other basic estimates (e.g. regression coefficient) AND variation (e.g. standard deviation) or associated estimates of uncertainty (e.g. confidence intervals) |
| ☒ | ☐ | For null hypothesis testing, the test statistic (e.g. *F*, *t*, *r*) with confidence intervals, effect sizes, degrees of freedom and *P* value noted<br>*Give P values as exact values whenever suitable.* |
| ☒ | ☐ | For Bayesian analysis, information on the choice of priors and Markov chain Monte Carlo settings |
| ☒ | ☐ | For hierarchical and complex designs, identification of the appropriate level for tests and full reporting of outcomes |
| ☒ | ☐ | Estimates of effect sizes (e.g. Cohen's *d*, Pearson's *r*), indicating how they were calculated |

*Our web collection on statistics for biologists contains articles on many of the points above.*

## Software and code

Policy information about availability of computer code

| | |
|---|---|
| Data collection | Cryo-EM data collection was automated using the Leginon data collection software (Suloway et al. J Struct Biol, 2005). |
| Data analysis | Kinetic data were analyzed using GraphPad Prism 8 (GraphPad Software, Inc.)<br>Mass Photometry data were analyzed using DiscoverMP (v2.3.0) software (Refeyn Ltd, Oxford, UK).<br>Sequence alignments were done using the Molecular Evolutionary Genetics Analysis (MEGA 7.0) software (Kumar et al. Mol Biol Evol, 2016).<br>Cryo-EM data collection was automated using the Leginon (v3.3) data collection software (Suloway et al., J. Struct. Biol., 2005).<br>Cryo-EM data were analyzed using Warp v1.0.7 (Tegunov & Cramer, Nat Methods, 2019), CryoSPARC v2.13 (Punjani et al., Nat. Methods, 2017), MotionCor2 (Zheng et al., Nat. Methods, 2017), RELION v3.1 (Zivanov et al., Elife, 2018), Gctf v1.06 (Zhang, J. Struct. Biol., 2016), and the the 3D FSC server (Tan et al., Nat Methods, 2017).<br>Model building and refinement were performed using Coot v0.9 and Phenix v1.20.1 (Adams et al., Acta Crystallogr D Biol Crystallogr., 2010; Emsley et al., Acta Crystallogr D Biol Crystallogr., 2010), and ERRASER through the Rosetta Online Server (https://rosie.graylab.jhu.edu/). Ideal geometry, secondary structure, and Geman-McClure distance restraints were generated in ProSMART v0.8 (Nicholls et al., Acta Crystallogr. D Biol. Crystallogr., 2012). MolProbity (Williams et al., Protein Sci., 2018) was used to assess the quality of the final structural model.<br>Structures were visualized and analyzed in ChimeraX (Goddard et al., Protein Sci., 2018). |

For manuscripts utilizing custom algorithms or software that are central to the research but not yet described in published literature, software must be made available to editors and reviewers. We strongly encourage code deposition in a community repository (e.g. GitHub). See the Nature Portfolio guidelines for submitting code & software for further information.

## Data

Policy information about availability of data

All manuscripts must include a data availability statement. This statement should provide the following information, where applicable:

- Accession codes, unique identifiers, or web links for publicly available datasets
- A description of any restrictions on data availability
- For clinical datasets or third party data, please ensure that the statement adheres to our policy

All data generated or analyzed during this study are included in this published article (and its supplementary information files). The cryo-EM map of mSerRS-mtRNASer(UGA) has been deposited in the Electron Microscopy Data Bank (EMDB) under the accession code EMD-29070. Atomic coordinates of the model have been deposited in the Protein Data Bank (PDB) under accession code 8FFY. The atomic coordinates used for molecular replacement or structural comparison were downloaded from the PDB: 1SER, 3L0U, 4TRA, 5UD5, 6YDP, 6ZM6, 7ONU, 7TZB, 7U2A, 7U2B. All tRNA gene sequences were retrieved from the tRNAdb/mitotRNAdb (http://trna.bioinf.uni-leipzig.de/), genomic tRNA database (GtRNAdb; http://gtrnadb.ucsc.edu/) or the National Center for Biotechnology Information database (NCBI; NC027264). Source data are provided with this paper.

## Research involving human participants, their data, or biological material

Policy information about studies with human participants or human data. See also policy information about sex, gender (identity/presentation), and sexual orientation and race, ethnicity and racism.

| | |
|---|---|
| Reporting on sex and gender | Does not apply. |
| Reporting on race, ethnicity, or other socially relevant groupings | Does not apply. |
| Population characteristics | Does not apply. |
| Recruitment | Does not apply. |
| Ethics oversight | Does not apply. |

Note that full information on the approval of the study protocol must also be provided in the manuscript.

# Field-specific reporting

Please select the one below that is the best fit for your research. If you are not sure, read the appropriate sections before making your selection.

☒ Life sciences ☐ Behavioural & social sciences ☐ Ecological, evolutionary & environmental sciences

For a reference copy of the document with all sections, see nature.com/documents/nr-reporting-summary-flat.pdf

# Life sciences study design

All studies must disclose on these points even when the disclosure is negative.

| | |
|---|---|
| Sample size | Sample sizes were not predetermined using statistical methods. For cryo-EM structure determination, a total of 3448 movies were collected for the tRNASer(UGA) dataset. The resulting particle stacks contained 2.7M particles. For cryo-EM analysis, the sample size was governed by the quality of the resulting cryo-EM map and was considered adequate when a sufficiently high quality of the cryo-EM map was obtained to perform structural fitting and atomic model building. The in vitro kinetic experiments were repeated three times to demonstrate reproducibility and to allow appropriate estimates of error by statistical analysis. |
| Data exclusions | In cryo-EM analysis, micrographs with ice were excluded. Particles belonging to 2D/3D classes that did not contain high-resolution structural features were discarded and not used in the final 3D reconstruction. No data were excluded from biochemical analyses. |
| Replication | Cryo-EM analysis was performed once, with 2.7M particles picked from a total of 3448 movies. Structural refinement was performed for multiple rounds with distinct sets of particles, resulting in the same density maps. In vitro biochemical experiments were repeated three times with consistent results. Purification of wild-type mSerRS was repeated two times (n=2), yielding similar and reproducible aminoacylation activities from each preparation. |
| Randomization | This study did not include treatment groups. |
| Blinding | Due to the nature of the project (understand molecular function of human mitochondrial aaRS/tRNA systems), investigators were not blinded and were aware of the sequence characteristics of all proteins/tRNAs used in the reported experiments. |

# Reporting for specific materials, systems and methods

We require information from authors about some types of materials, experimental systems and methods used in many studies. Here, indicate whether each material, system or method listed is relevant to your study. If you are not sure if a list item applies to your research, read the appropriate section before selecting a response.

## Materials & experimental systems

| n/a | Involved in the study |
|---|---|
| ☒ | ☐ Antibodies |
| ☒ | ☐ Eukaryotic cell lines |
| ☒ | ☐ Palaeontology and archaeology |
| ☒ | ☐ Animals and other organisms |
| ☒ | ☐ Clinical data |
| ☒ | ☐ Dual use research of concern |
| ☒ | ☐ Plants |

## Methods

| n/a | Involved in the study |
|---|---|
| ☒ | ☐ ChIP-seq |
| ☒ | ☐ Flow cytometry |
| ☒ | ☐ MRI-based neuroimaging |
