# Supplementary material for: Structural basis for a degenerate tRNA identity code and the evolution of bimodal specificity in human mitochondrial tRNA recognition
Source: Nat Commun. 2023 Aug 9;14:4794. doi: 10.1038/s41467-023-40354-2 (PMC10412605; doi:10.1038/s41467-023-40354-2)
Supplement: Supplementary file 3 — Peer Review [file 41467_2023_40354_MOESM3_ESM.pdf]

# Peer Review File

## Structural basis for a degenerate tRNA identity code and the evolution of bimodal specificity in human mitochondrial tRNA recognition

**Open Access** This file is licensed under a Creative Commons Attribution 4.0 International License, which permits use, sharing, adaptation, distribution and reproduction in any medium or format, as long as you give appropriate credit to the original author(s) and the source, provide a link to the Creative Commons license, and indicate if changes were made. In the cases where the authors are anonymous, such as is the case for the reports of anonymous peer reviewers, author attribution should be to 'Anonymous Referee' followed by a clear attribution to the source work. The images or other third party material in this file are included in the article's Creative Commons license, unless indicated otherwise in a credit line to the material. If material is not included in the article's Creative Commons license and your intended use is not permitted by statutory regulation or exceeds the permitted use, you will need to obtain permission directly from the copyright holder. To view a copy of this license, visit http://creativecommons.org/licenses/by/4.0/.

REVIEWER COMMENTS

Reviewer #1 (Remarks to the Author):

Structural basis for a degenerate tRNA identity code and the evolution of bimodal specificity in human mitochondrial tRNA recognition

Kuhle, Hirschi, Doerfel, Lander & Schimmel

In this interesting manuscript the authors complement their previous structural analysis of the recognition between human

mSerRS and tRNA-Ser(GCU) with the equivalent with tRNA-Ser(UGA).

Because these two tRNAs are extremely different, the mechanism

that allows mSerRS to recognize both substrates has been a long standing question. Remarkably, this paper shows that both substrates bind mSerRS through the same binding surface through a combination of individual interactions that avoids specific sequence recognition by interacting with the tRNA backbone but, to a large extent, relying in the same protein domains.

This fascinating result finally clarifies this long standing riddle, while describing a surprising mechanism for recognition adaptation.

The manuscript reads extremely well, and my only minor comments

in terms of the language is that the authors should avoid the use

of the word 'homology' in lines 133, 139, and 153, and substitute

it by 'similarity', or 'identity', as homology is a qualitative term that cannot be 'highest' or 'lowest'.

My main concern is with the interpretation of the described phenomenon as the effect of Muller's ratchet acting on tRNA genes that is met by adaptations in mSerRS. The incidence of Muller's ratchet in mitochondria is a controversial because the existence of compensatory mutations in mitochondrial genomes has been reported (Howe & Denver 2008), and because the deleterious drift

in mitochondrial genomes is in constant interplay with the evolution of the nuclear-encoded mitochondrial proteins. It is possible that the reduction of size of mitochondrial genomes may be due to Muller's ratchet, but the changes in highly interactive molecules (and essential ones, too) such as tRNAs must be under a strong selective pressure to avoid the accumulation of deleterious mutations. In that regard, it has been proposed that

mitochondrial tRNAs may be rapidly drifting because their reduction in number causes a subsequent reduction in their recognition constraints that would allow them, for example, to shift identity elements otherwise invariable (as in the case of tRNA-Ala), or even allow changes in the genetic code often organelles. Perhaps the authors could consider this theory as another possible explanation for the large divergence between mitochondrial tRNAs.

Finally, the authors also structure all their discussion around the notion that it was mSerRS who evolved to be flexible enough

to recognize these very different tRNAs. A more 'tRNA-centric'

possibility is that the drift of the tRNAs was always restricted

to tRNA sequences that would continue to take advantage of the

existing 'anchors' in mSerRS. In other words, the tRNAs drifted,

yes, but never abandoned a sequence space that could bind effectively to the available mSerRS surface.

A way to distinguish between these two possibilities would be

to ask whether the tRNA-binding surfaces in mammalian mSerRS are

conserved or not with mSerRSs from organisms whose mitochondrial tRNA-Ser are more canonical in nature. If the mSerRS sequences are relatively invariant, I would favor the alternative hypothesis.

Reviewer #2 (Remarks to the Author):

Structural basis for a degenerate tRNA identity code and the evolution of bimodal specificity in human mitochondrial tRNA recognition by Kuhle et al.

The mitochondrial SARS2 can charge two different tRNAs that have diverse structures: An oddly Y-shaped tRNA(Ser-GCU) and tRNA(Ser-UGA) with a more or less normal tRNA cloverleaf structure but with a reduced variable loop. The submitted ms is a follow-up to the authors' recent paper on the structure of the complex between SARS2 and tRNA(Ser-GCU) (Kuhle et al., Nat. Com. 2022).

Using the same approaches as in their recently published paper (the M&M section is ~95% identical), they now determine the structure of SARS2 bound to tRNA(Ser-UGA) and perform some mutagenesis

with accompanying assays (of which most mutations have already been tested previously for the human or bovine system, but yes, it is always good to verify).

Furthermore, as already implied in previous publications based on results from mutagenesis, biochemical assays, and foot printing, the new structure shows that the two tRNA species share the binding interface to SARS2 and interact almost exclusively with SARS2 through their phosphate-sugar backbone. There is slightly more “caressing” of the arm-elbow interface by the SARS2 helical arm in the case of tRNA(Ser-UGA), but that is to be regarded as a very minor enzyme-substrate adaption (Fig 5a,b).

Nevertheless, the structure, even if not resolved to as high resolution as is more or less common nowadays, is competently done, especially given the small particle size and preferential orientation tendency.

1. Just as in the case of tRNA(Ser-GCU), only one protomer in the SARS2 dimer binds a tRNA while a SerSA ligand is found in both. This is understandable from the fact that the amount of tRNA added in the preparation of the complex is sub-stoichiometric (1.5x tRNA to 2 active sites/binding interfaces). There is a section in the M&M part describing how an active site titration was done. Still, the results from this assay are not given in the results section of the paper or touched upon in the discussion section, especially since it is contradicted by previous work by the Watanabe group using gel-retardation assays with mature (modified) tRNA purified from tissue (JBC (2001), 276, 50, p 46770–, Fig. 2B). This should be addressed.

2. It is shown in the same work from the Watanabe group that Kcat/Km for native tRNA(Ser-UGA) is 11 times higher than for in-vitro transcribed tRNA. This should be addressed and analyzed in light of the present structure. After all, what matters is the tRNA(Ser-UGA) in the true environment of mammalian mitochondria in contrast to the T7 transcripts used for the structural work and biochemistry presented in the submitted ms.

3. On p. 6 line 118 there is the following speculation “The distinct evolutionary origins of the two tRNAs imply that their shared non-canonical features are the result of convergent evolution.“. This is far from certain. Mitochondrial tRNAs should, by necessity, evolve along epistatic ridges in the fitness landscape. The process of evolving to bind a new aminoacyltransferase enzyme is likely to involve a deep fitness valley, as the tRNA would need to overcome the selective pressures that have shaped its current structure and function. In contrast, an ancestral tRNAs charged by an ancestral SARS2 would be able to evolve with small changes (and their compensatory mutations, if advantageous) in its sequence/structure that, over time, and while retaining the binding interface, diverge to the present UGA/GCU variants that still both are successfully charged by SARS2 thereby avoiding evolutionary detours through deep fitness valleys. These divergent evolutionary trajectories could arise due to different selective pressures acting on each tRNA. In this case, the relatively slower charging of

tRNA(Ser-GCU) compared to tRNA(Ser-UGA) provides, for example, opportunities to regulate the translational pace to match folding and membrane-insertion requirements for the mitochondrially-encoded components in the OXPHOS system.

4. When it comes to the T-stem wobbles, the authors find the same very marginal effect on G50-U64, G51:U63 as others many years before them but do not test, or even discuss, the effect of a U65, which would arguably be the most interesting test for the importance of this region given the high level of conservation for the GC WC-base pair (CG in the tRNA(Ser-GCU) case; Fig. 3A). This position should be tested experimentally.

5. On p. 10, starting on line 201. "This suggests that the contribution of the G:U wobble pairs in the T-stem interface is idiosyncratic to mtRNASer(UGA) recognition, and may provide a mechanistic rationale for the markedly distinct effects the removal of the C-tail (deleting 15 residues from the C-terminus) has on the kcat and Km between bovine mSerRS and mtRNASer(UGA) versus mtRNASer(GCU)24. " But Ref. 24 shows that deleting the 15 residues in the C-term barely affects the charging rate of tRNA (Ser-UGA). Please clarify this passage and provide firm arguments using the new structural and previously published biochemical data in Ref. 24.

6. Lastly, the ms contains long sections on evolution, how mechanisms coupled to Müllers ratchet risk degenerate the mitochondrial genome, and also promise in the introduction:

"insight into the evolution and functional properties of an essential cellular machinery exposed to high mutation pressure and random genetic drift. They highlight an underlying evolutionary dynamic in which biological innovation is driven by the pressure to maintain indispensable molecular functions under conditions of mutational disruption.".

An even higher pitch is used at the end of the discussion:

"our analysis highlights an evolutionary dynamic in which the mutational divergence of mtRNASer(UGA) and mtRNASer(GCU) along distinct evolutionary trajectories resulted in a radical rewiring of the intermolecular recognition rules underlying mitochondrial genetic code expression. Our data demonstrate how the need to maintain an indispensable molecular function in response to mutational disruption can drive fundamental biological innovation even in the most conserved cellular processes, with multiple evolutionary pathways leading to functionally equivalent outcomes."

Not only are these formulations quite hyperbolic, they are also unnecessary in a paper of this type and should be trimmed and toned down. Especially so, given the actual content of the paper, the fact that there is a strong purifying selection in the germline and the relatively high evolutionary rate of

mitochondrial tRNAs does not inevitably lead to a mutational meltdown in part for reasons linefeed in p.3, above.

Minor comments:

p. 15, 302: show, not shows (parallels).

Table S1: keV is not a unit for voltage but for energy; likewise, on p.20, 404 in M&M.

Reviewer #3 (Remarks to the Author):

The manuscript of Kuhle et al. elucidates the different binding modes of mitochondrial serine aminoacyl-tRNA synthetase (mSerRS) with two mtRNASer isoacceptors. The study represents a follow-up study of their previous publication (Kuhle et al., Nat. Commun. 2022), where they have analyzed the structure of mSerRS with mtRNASer(GCU). In the current study, they solved the structure of mSerRS with mtRNASer(UGA) and compare it with their previous structure. Overall, the manuscript is well-written, nicely structured, and easy to follow. However, the amount of new knowledge gained from this study is limited compared to their previous study and previous biochemical work (Shimada et al., JBC 2001).

Furthermore, this reviewer is concerned about the biological relevance of the study as the authors use in vitro transcribed tRNAs, which lack post-transcriptional modifications. Even though the authors analyze in great detail the structure of mSerRS with tRNASer(UGA), they neglect the fact that some of the residues analyzed are post-transcriptionally modified in vivo. This mainly concerns the T-arm, where three modifications have been identified previously (Suzuki et al., Nat. Commun. 2020). The T-arm is a main contributor to the mSerRS interaction, and all modification sites seem to be participating in this interaction. Notably, these residues are highly conserved in the 41 primate species analyzed in the manuscript (Figure 3a), which supports their importance. Kuhle et al. compare this region structurally to its isoacceptor and identify differences, which are presented as major findings in the manuscript (Figure 3c and d, 4d, 5 a-c, 6a and b). Still, the potential effect of modifications is not considered.

Other comments:

• In lines 177-179, the authors write:

“To bind mtRNASer(UGA), mSerRS involves 23 residues that are nearly identical to those involved in GCU isoacceptor binding.“

Yet, the authors write in lines 23+24:

“We find that despite their common function, mtRNASer(UGA) and mtRNASer(GCU) show no constrain to converge on a shared structure or sequence motif for interactions with mSerRS.”

Therefore, the similarities and differences of both binding modes are not clear. Are there any structural differences in the binding modes besides the T-arm region?

• In Shimada et al. (JBC 2001) it was demonstrated that mSerRS can misacylate mitochondrial tRNAGln due to the same sequence in the T-arm. It would be interesting to compare the structure of mtRNASer(UGA) and mtRNAGln bound to mSerRS or at least discuss it in the text.

**POINT-BY-POINT RESPONSE**

**Reviewer #1 (Remarks to the Author):**

Structural basis for a degenerate tRNA identity code and the evolution of bimodal specificity in human mitochondrial tRNA recognition Kuhle, Hirschi, Doerfel, Lander & Schimmel

In this interesting manuscript the authors complement their previous structural analysis of the recognition between human mSerRS and tRNA-Ser(GCU) with the equivalent with tRNA-Ser(UGA). Because these two tRNAs are extremely different, the mechanism that allows mSerRS to recognize both substrates has been a long standing question. Remarkably, this paper shows that both substrates bind mSerRS through the same binding surface through a combination of individual interactions that avoids specific sequence recognition by interacting with the tRNA backbone but, to a large extent, relying in the same protein domains. This fascinating result finally clarifies this long standing riddle, while describing a surprising mechanism for recognition adaptation.

The manuscript reads extremely well, and my only minor comments in terms of the language is that the authors should avoid the use of the word 'homology' in lines 133, 139, and 153, and substitute it by 'similarity', or 'identity', as homology is a qualitative term that cannot be 'highest' or 'lowest'.

Thank you for pointing this out. We changed the wording accordingly.

My main concern is with the interpretation of the described phenomenon as the effect of Muller's ratchet acting on tRNA genes that is met by adaptations in mSerRS. The incidence of Muller's ratchet in mitochondria is a controversial because the existence of compensatory mutations in mitochondrial genomes has been reported (Howe & Denver 2008), and because the deleterious drift in mitochondrial genomes is in constant interplay with the evolution of the nuclear-encoded mitochondrial proteins. It is possible that the reduction of size of mitochondrial genomes may be due to Muller's ratchet, but the changes in highly interactive molecules (and essential ones, too) such as tRNAs must be under a strong selective pressure to avoid the accumulation of deleterious mutations. In that regard, it has been proposed that mitochondrial tRNAs may be rapidly drifting because their reduction in number causes a subsequent reduction in their recognition constraints that would allow them, for example, to shift identity elements otherwise invariable (as in the case of tRNA-Ala), or even allow changes in the genetic code often organelles. Perhaps the authors could consider this theory as another possible explanation for the large divergence between mitochondrial tRNAs.

Thank you for bringing up this important point. We agree that there are likely other factors acting on mitochondrially encoded genes besides Muller's ratchet, including relaxed selective pressure on mt-tRNA genes. We would like to point out, however, that the mutational erosion of mt-tRNAs is not equally strong across eukaryotes (although the overall number of mt-tRNA genes remains essentially unchanged) but is significantly more dramatic in animals than in non-animal species. We would therefore argue that, while mt-tRNAs in general are under relaxed functional constraints, animal mt-tRNAs, in addition, are exposed to elevated mutation pressure and an increased strength of Muller's ratchet. We would therefore not consider these as alternative explanations, but rather as two complementary factors to explain the observed rapid evolution of animal mt-tRNAs.

We addressed this point in an additional paragraph in the discussion section of the revised manuscript (p. 14).

Finally, the authors also structure all their discussion around the notion that it was mSerRS who evolved to be flexible enough to recognize these very different tRNAs. A more 'tRNA-centric' possibility is that the drift of the tRNAs was always restricted to tRNA sequences that would continue to take advantage of the existing 'anchors' in mSerRS. In other words, the tRNAs drifted, yes, but never abandoned a sequence space that could bind effectively to the available mSerRS surface. A way to distinguish between these two possibilities would be to ask whether the tRNA-binding surfaces in mammalian mSerRS are conserved or not with mSerRSs from organisms whose mitochondrial tRNA-Ser are more canonical in nature. If the mSerRS sequences are relatively invariant, I would favor the alternative hypothesis.

Thank you for raising this point. With regard to the conservation of tRNA-binding surfaces, we did analyze the sequences of non-bilaterian mSerRSs, including those from Porifera and the Placozoan *Trichoplax adhaerens* (*Ta*), which are the closest relatives to bilaterians that still encode both mtRNA$^{Ser}$ isoacceptors with canonical cloverleaf and extended V-arm. As we show in Supplementary Figure S8 and the additional Supplementary Figure S9, some of the characteristics of the bilaterian mSerRS interface are indeed emerging in Porifera and Placozoa. However, it is important to note that, although occupied by a positively charged side chain, neither Lys 110, nor Arg146 are invariant in these taxa, and the C-tail is completely missing. A rudimentary N-helix may be present, but it is very different in sequence to the corresponding region in chordates and could also be part of the MTS. Thus, although the new tRNA interface is indeed beginning to emerge in non-bilaterians, it seems that the evolution and fixation of the full set of 'anchor' elements for mtRNAs in mSerRS occurred within the bilaterian lineage (i.e. after the split from Placozoa) and their occurrence is invariably linked to a degenerate set of mtRNA$^{Ser}$ isoacceptors. At the same time, we agree that the mtRNA$^{Ser}$ sequences and in particular their structures are likely restricted by the available 'anchor points' in mSerRS once the bimodal recognition mechanism had been established at some point in the course of bilaterian animal evolution. However, its chronology, i.e. whether the evolution of the anchor points preceded the divergence of mtRNA$^{Ser}$ recognition elements or vice versa, remains unclear at present and will require further investigation, in particular into the mSerRS-mtRNA$^{Ser}$ interactions in bilaterian taxa other than vertebrates, where they appear to be more complex [Guitart et al., JBC, 2010; Picchioni et al., Sci. Adv. 2019]. Given these uncertainties, we would prefer to maintain the focus of our current discussion on the mammalian/vertebrate lineage. A more detailed analysis and presentation of recognition elements in mSerRSs and tRNAs across animals were included in the new Supplementary Fig. 9. We hope the reviewer agrees with this decision.

**Reviewer #2 (Remarks to the Author):**

Structural basis for a degenerate tRNA identity code and the evolution of bimodal specificity in human mitochondrial tRNA recognition by Kuhle et al.

The mitochondrial SARS2 can charge two different tRNAs that have diverse structures: An oddly Y-shaped tRNA(Ser-GCU) and tRNA(Ser-UGA) with a more or less normal tRNA cloverleaf structure but

with a reduced variable loop. The submitted ms is a follow-up to the authors' recent paper on the structure of the complex between SARS2 and tRNA(Ser-GCU) (Kuhle et al., Nat. Com. 2022).

Using the same approaches as in their recently published paper (the M&M section is ~95% identical), they now determine the structure of SARS2 bound to tRNA(Ser-UGA) and perform some mutagenesis with accompanying assays (of which most mutations have already been tested previously for the human or bovine system, but yes, it is always good to verify).

Given the unusual characteristics of animal mSerRS-mtRNA recognition systems (when compared to their canonical (cytosolic) counterparts) and elevated mutation rates in animal mSerRS and mtRNA(Ser) isoacceptors, it was by no means certain that the recognition mechanism or the nucleotides and amino acids involved should be identical across the mammalian lineage, including humans. Our biochemical results for the human system are therefore evidence for the conservation of this unusual mSerRS-mtRNA recognition system across mammals (and possibly beyond), rather than just a verification of the results obtained in *B. taurus*. (We are not aware of an earlier study on the human system which – as implied by the reviewer – tested the same mutations that we tested here for the mSerRS-mtRNA$^{Ser(UGA)}$ interaction.)
Also, it should be noted in this context that not all the mSerRS residues that were suggested to play a role in mtRNA$^{Ser}$ binding according to the mutational studies by Chimnaronk et al. [The EMBO Journal, 2005] actually contact the mtRNAs directly; for example, the side chains of Arg32, Tyr28, and Arg24 lie at a distance of 7, 9, and 13 Å from the tRNA. Similarly, it was suggested on the basis of mutational analyses of bovine mtRNA that mSerRS specifically recognizes sequence elements in each mtRNA$^{Ser}$ [Shimada et al., JBC, 2001]. Our analysis shows that these elements are in fact not accessible to direct interactions with mSerRS. Finally, based on their results, Chimnaronk et al. and Shimada et al. come to contradictory conclusions, deducing either indirect structure-based readout [Chimnaronk et al., The EMBO Journal, 2005] or sequence-specific recognition [Shimada et al., JBC, 2001]. Clear evidence supporting one interpretation over the other had not been provided by either of the two papers. Our present study clarifies this question at least for the human system, supporting an indirect, structure-based readout.

Furthermore, as already implied in previous publications based on results from mutagenesis, biochemical assays, and foot printing, the new structure shows that the two tRNA species share the binding interface to SARS2 and interact almost exclusively with SARS2 through their phosphate-sugar backbone. There is slightly more "caressing" of the arm-elbow interface by the SARS2 helical arm in the case of tRNA(Ser-UGA), but that is to be regarded as a very minor enzyme-substrate adaption (Fig 5a,b).

Nevertheless, the structure, even if not resolved to as high resolution as is more or less common nowadays, is competently done, especially given the small particle size and preferential orientation tendency.

1. Just as in the case of tRNA(Ser-GCU), only one protomer in the SARS2 dimer binds a tRNA while a SerSA ligand is found in both. This is understandable from the fact that the amount of tRNA added in the preparation of the complex is sub-stoichiometric (1.5x tRNA to 2 active sites/binding interfaces). There is a section in the M&M part describing how an active site titration was done. Still, the results

from this assay are not given in the results section of the paper or touched upon in the discussion section, especially since it is contradicted by previous work by the Watanabe group using gel-retardation assays with mature (modified) tRNA purified from tissue (JBC (2001), 276, 50, p 46770–, Fig. 2B). This should be addressed.

Thank you for raising this point. First, we would like to point out that the complex was not prepared using sub-stoichiometric amounts of tRNA. As we describe in the M&M section (p. 21): "The mSerRS-SerSA complex was then mixed with mtRNA$^{Ser(UGA)}$ at a protein:tRNA molar ratio of 1:1.5 (corresponding to a 3-fold molar excess of tRNA over mSerRS dimer)…". Thus, there was 1.5-fold tRNA for each binding site on the mSerRS dimer. We tested higher excess of tRNA over mSerRS (up to 5-fold), which, however, did not result in an increased occupancy of the mSerRS dimer with tRNA (for both mtRNA(Ser) isoacceptors).
To further test the stoichiometry of the human mSerRS-mtRNA$^{Ser(UGA)}$ complex, we used mass photometry which enables the accurate mass measurement of single molecules and complexes in solution at low concentrations [Young et al, Science, 2018]. As shown in the figure below, the addition of a 5-fold excess of mtRNA$^{Ser(UGA)}$ (125 nM) to mSerRS (25 nM) results in the formation of a single peak shifted by 21 kDa relative to the free mSerRS dimer, corresponding to a single mtRNA$^{Ser(UGA)}$ (MW = 23.7 kDa). Thus, mass photometry supports a 2:1 ratio of mSerRS to mtRNA$^{Ser(UGA)}$ at these concentrations, which is consistent with our earlier results for the mSerRS-mtRNA$^{Ser(GCU)}$ complex [Kuhle et al., Nat. Com., 2022]. We included the new data and methods in the revised manuscript (p. 5 line 98, p. 20 line 418, and Supplementary Fig. 3b).
Why our cryo-EM and mass photometry results differ from those reported by Shimada et al. [Shimada et al, JBC, 2001] we do not know. Possible reasons are the relatively high enzyme and tRNA concentrations used by Shimada et al (440 nM mSerRS and up to 5 µM mtRNA$^{Ser}$) and the use of EMSA to determine complex formation, which is known to stabilize complexes by caging effects. It may also be important to note that Shimada et al. use the bovine system, raising the possibility of species-specific differences in the stability of 2:2 complexes. Finally, post-transcriptional modifications may indeed play a role in stabilizing a 2:2 complex, as suggested by the reviewer.
Yet, we would also like to point out that structural and biochemical information available from non-mitochondrial SerRSs likewise suggest that the SerRS dimer preferably binds only one tRNA [Yaremchuk et al, FEBS, 1992; Biou et al, Science, 1994; Cusack et al, EMBO J, 1996], which led Biou et al. to suggest that "this stoichiometry might have a particular biological significance" [Biou et al, Science, 1994]. This is consistent with studies suggesting that the two tRNA$^{Ser}$ binding sites of SerRSs are not equivalent [Krauss et al, Eur. J. Biochem., 1975; Rigler et al, Eur. J. Biochem., 1976; Wang et al, NAR, 2015]. The reasons for this non-equivalence are not known – even for canonical SerRSs. Unfortunately, the quality of the EM density in the corresponding regions of the model (with the second helical arm not defined in the density) is not sufficiently high to provide a detailed mechanistic explanation. In this context, it may also be important to keep in mind that we have no empirical information regarding the absolute or even relative concentrations of mt-aaRSs and mtRNAs in mammalian mitochondria, which would be important to understand the biological relevance of a 2:1 vs. 2:2 stoichiometry. We therefore chose to not include a (necessarily speculative) discussion of the mechanistic basis or biological relevance for this asymmetry, especially as it is not the focus of this manuscript. We hope that the reviewer agrees with our decision. We included a brief discussion of the stoichiometry of the complex in the revised manuscript, together with the new mass photometry results (p. 5 line 98 and Supplementary Fig. 3b).

Regarding the active site titrations: This is a standard assay that is performed in our lab to determine the 'number of active sites' in protein preparations. It monitors the formation of the seryl-adenylate and is performed in the absence of tRNA. It does not provide information that is immediately relevant to the main arguments of the manuscript and was therefore not included. Below, we provide the active site titration for two of our mSerRS preparations, which shows that >80% of the purified mSerRSs contain catalytically competent active sites.

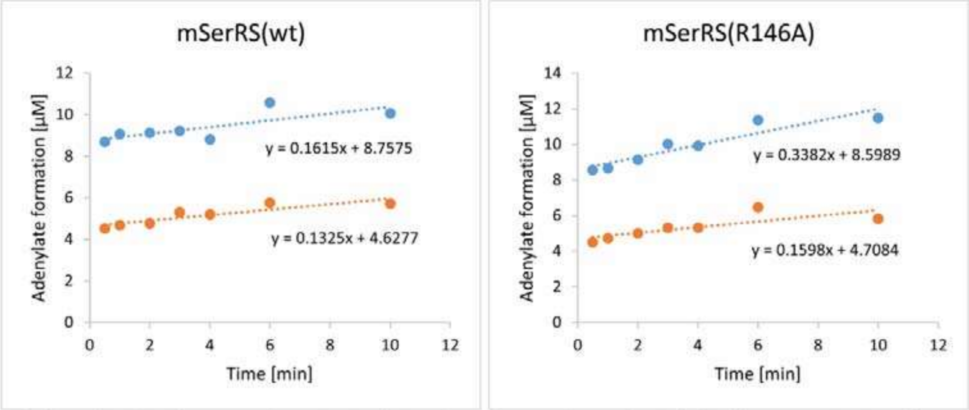


Active site titration experiments to determine the number of catalytically competent active sites for two preparations of mSerRS(wt) and mSerRS(R146A). Reactions contained either 5 µM (orange) or 10 µM (blue) of purified human mSerRS, 20 mM L-serine, 22 nM [γ-32P]-ATP, in assay buffer (100 mM HEPES pH 7.5, 20 mM KCl, 10 mM MgCl2, 2 mM DTT, and 2 mg/mL pyrophosphatase) and were carried out at room temperature.

2. It is shown in the same work from the Watanabe group that Kcat/Km for native tRNA(Ser-UGA) is 11 times higher than for in-vitro transcribed tRNA. This should be addressed and analyzed in light of the present structure. After all, what matters is the tRNA(Ser-UGA) in the true environment of mammalian mitochondria in contrast to the T7 transcripts used for the structural work and biochemistry presented in the submitted ms.

We agree with the reviewer that this is an important point that should have been addressed from the start. We included a discussion in the revised manuscript on p. 6 line 112 and p. 12 line 239.

3. On p. 6 line 118 there is the following speculation "The distinct evolutionary origins of the two tRNAs imply that their shared non-canonical features are the result of convergent evolution.". This is far from certain. Mitochondrial tRNAs should, by necessity, evolve along epistatic ridges in the fitness landscape. The process of evolving to bind a new aminoacyltransferase enzyme is likely to involve a deep fitness valley, as the tRNA would need to overcome the selective pressures that have shaped its current structure and function. In contrast, an ancestral tRNAs charged by an ancestral SARS2 would be able to evolve with small changes (and their compensatory mutations, if advantageous) in its sequence/structure that, over time, and while retaining the binding interface, diverge to the present UGA/GCU variants that still both are successfully charged by SARS2 thereby avoiding evolutionary detours through deep fitness valleys. These divergent evolutionary trajectories could arise due to different selective pressures acting on each tRNA. In this case, the relatively slower charging of tRNA(Ser-GCU) compared to tRNA(Ser-UGA) provides, for example, opportunities to regulate the

translational pace to match folding and membrane-insertion requirements for the mitochondrially-encoded components in the OXPHOS system.

This appears to be a misunderstanding arising from a lack of clarity on our part. The cited passage on p.6 line 118 refers to the structural similarities observed between $mtRNA^{Ser(UGA)}$ and the cytosolic $tRNA^{Pyl}$ found in some bacteria and archaea – and not to a comparison between $mtRNA^{Ser(UGA)}$ and $mtRNA^{Ser(GCU)}$. We reworded the corresponding passage in the revised manuscript (p. 7 line 124 in the revised manuscript).

4. When it comes to the T-stem wobbles, the authors find the same very marginal effect on G50-U64, G51:U63 as others many years before them but do not test, or even discuss, the effect of a U65, which would arguably be the most interesting test for the importance of this region given the high level of conservation for the GC WC-base pair (CG in the tRNA(Ser-GCU) case; Fig. 3A). This position should be tested experimentally.

Our focus on the wobble pairs was motivated by the fact that 1.) G50:U64 forms the only potentially base-specific interaction with the synthetase and 2.) they are conserved despite having a potentially distorting/destabilizing effect on the local structure of the T-stem. No base-specific interactions are formed with G49:C65 in $mtRNA^{Ser(UGA)}$ or the C49:G65 in $mtRNA^{Ser(GCU)}$, providing no clear argument to assume that it functions as a positive identity element beyond its role to stabilize the base of the T-stem. However, the main reason for us to omit the G49:U65 mutation was that it causes two distinct tRNA conformations according to native PAGE, either by destabilizing the native fold or by stabilizing an alternative structure:

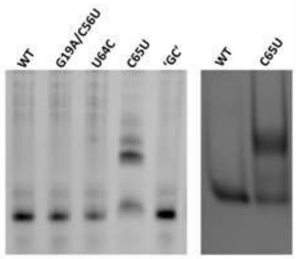


Native PAGE (left) and TAE-agarose gel (right) for wild-type and mutant $mtRNA^{Ser(UGA)}$ variants. The C65U mutant forms a dominant alternative conformation migrating above the major form of all other variants.

We included a discussion of the effects of the U65 mutant that was reported for the bovine system and its potential role as negative recognition element in bovine $mtRNA^{Gln}$ as suggested in Shimada et al., JBC, 2001. (p. 11 line 218 and on p. 12 line 243).

5. On p. 10, starting on line 201. "This suggests that the contribution of the G:U wobble pairs in the T-stem interface is idiosyncratic to mtRNASer(UGA) recognition, and may provide a mechanistic rationale for the markedly distinct effects the removal of the C-tail (deleting 15 residues from the C-terminus) has on the kcat and Km between bovine mSerRS and mtRNASer(UGA) versus mtRNASer(GCU)24. " But Ref. 24 shows that deleting the 15 residues in the C-term barely affects the charging rate of tRNA (Ser-UGA). Please clarify this passage and provide firm arguments using the new structural and previously published biochemical data in Ref. 24.

In Ref. 24 p.3375 Chimnaronk et al. [The EMBO Journal, 2005] state that “the ΔC-11 mutant (i.e. deletion from Lys491) significantly decreased the serylation activity for both substrate tRNAs. It is surprising that the cloverleaf-shaped mt $tRNA^{Ser}_{UGA}$ is more susceptible to the length of the C-tail, as it requires Gln488 (ΔC-14 mutant) for reasonable serylation ability (the ΔC-16 mutant can still charge $tRNA^{Ser}_{GCU}$ by serine to some extent). […] Intriguingly, the kinetic parameters determination of the ΔC-15 mutant showed a 28-fold higher $K_m$ value than the WT enzyme for $tRNA^{Ser}_{GCU}$ (0.78 and 21.92 mM for the WT and the ΔC-15 mutant, respectively), while the $k_{cat}$ remained substantially unchanged. Conversely, the ΔC-15 mutant decreased the $k_{cat}$ by nine-fold ($12.1x10^{-3}$ and $1.3x10^{-3}$ $s^{-1}$ for the WT and the ΔC-15 mutant, respectively), whereas it barely affected the $K_m$ for $tRNA^{Ser}_{UGA}$.”

6. Lastly, the ms contains long sections on evolution, how mechanisms coupled to Müllers ratchet risk degenerate the mitochondrial genome, and also promise in the introduction: “insight into the evolution and functional properties of an essential cellular machinery exposed to high mutation pressure and random genetic drift. They highlight an underlying evolutionary dynamic in which biological innovation is driven by the pressure to maintain indispensable molecular functions under conditions of mutational disruption.”.

An even higher pitch is used at the end of the discussion:

“our analysis highlights an evolutionary dynamic in which the mutational divergence of mtRNASer(UGA) and mtRNASer(GCU) along distinct evolutionary trajectories resulted in a radical rewiring of the intermolecular recognition rules underlying mitochondrial genetic code expression. Our data demonstrate how the need to maintain an indispensable molecular function in response to mutational disruption can drive fundamental biological innovation even in the most conserved cellular processes, with multiple evolutionary pathways leading to functionally equivalent outcomes.”

Not only are these formulations quite hyperbolic, they are also unnecessary in a paper of this type and should be trimmed and toned down. Especially so, given the actual content of the paper, the fact that there is a strong purifying selection in the germline and the relatively high evolutionary rate of mitochondrial tRNAs does not inevitably lead to a mutational meltdown in part for reasons linefeed in p.3, above.

We are sorry the reviewer does not agree with the style in which these passages are written. However, in view of the question we address in this manuscript, the data presented, and their interpretation, they are neither exaggerated, nor factually wrong. Given the reviewer’s comments, we would like to clarify a few points: 1.) We think that there is no question about the fact that animal mtRNAs have undergone an unparalleled mutational divergence from their ancestral canonical forms: They are thermodynamically unstable, accumulated insertions, deletions, higher numbers of mismatches, have lost entire structural domains, lost canonical identity features, and are highly susceptible to disease-causing mutations. 2.) The gradual accumulation of slightly deleterious mutations is the most realistic explanation for the bulk of these changes in mtRNAs (rather than adaptive fine-tuning). 3.) The high rates of molecular evolution and divergence in animal mtRNAs occur regardless of the strong purifying selection in the animal germline. As has been pointed out previously, this is likely due to a wider selective sieve for mtRNA mutations [Lynch, Mol. Biol. Evol., 1997], consistent with comparatively weak purifying germline selection for mutations in mtRNAs (compared to non-synonymous mutations

in protein-coding genes) [Stewart et al., PLOS Biol., 2008] and the observation that mtRNA polymorphisms are frequent in recently derived mtDNA haplogroup variants in humans [Elson et al., Am. J. Hum. Genet., 2004]. 4.) Their evident mutational divergence would make most mtRNAs dysfunctional in the context of canonical translation machineries. (This is in particular true for the two mtRNA$^{Ser}$ variants our work focusses on.) 5.) Nowhere in this manuscript do we state that the high mutation rates of mtRNAs inevitably lead to their mutational meltdown. Instead, what we do state is that increased mutation rates and inefficient selection pose a risk of an irreversible loss of sequence information over time (point 1 above), which would be incompatible with canonical tRNA recognition systems (point 4). This leads us to ask how indispensable molecular interactions like those between aaRSs and tRNAs, which usually rely on the strong conservation of sequence information to maintain intermolecular specificity, **avoid** functional meltdown under such conditions. What we (in combination with earlier work on the bovine system) show is that the mammalian serylation system adapted to these conditions by radically reinventing the rules on which this intermolecular specificity is based. In other words, I) this work is in fact based on the premise that a mutational meltdown of mtRNAs is avoided, and II) it addresses the molecular mechanism by which this is achieved.

Minor comments:
p. 15, 302: show, not shows (parallels).
Thank you. We corrected the mistake.
Table S1: keV is not a unit for voltage but for energy; likewise, on p.20, 404 in M&M.
Thank you for catching this. keV was changed to kV on p. 20 (now p. 21) and in Table S1.

**Reviewer #3 (Remarks to the Author):**

The manuscript of Kuhle et al. elucidates the different binding modes of mitochondrial serine aminoacyl-tRNA synthetase (mSerRS) with two mtRNASer isoacceptors. The study represents a follow-up study of their previous publication (Kuhle et al., Nat. Commun. 2022), where they have analyzed the structure of mSerRS with mtRNASer(GCU). In the current study, they solved the structure of mSerRS with mtRNASer(UGA) and compare it with their previous structure. Overall, the manuscript is well-written, nicely structured, and easy to follow. However, the amount of new knowledge gained from this study is limited compared to their previous study and previous biochemical work (Shimada et al., JBC 2001).

The molecular mechanisms of how animal mtDNA-encoded genes avoid functional erosion by mutation accumulation is a long-standing question and object of an ongoing debate [e.g. see Hill, Trends Genet., 2020]. This debate remains largely limited to genetic analyses (e.g. genome-wide evolutionary rate analyses), as comprehensive insight into the molecular and functional properties of mtDNA-encoded genes and their interactions with nuclear-encoded binding partners is still lacking. The divergence and idiosyncratic erosion of mtDNA-encoded mtRNA$^{Ser(GCU)}$ and mtRNA$^{Ser(UGA)}$ (and the need for a nuclear-encoded mSerRS to maintain specificity with both) provides a unique opportunity to gain insight into these molecular and functional properties and the principles of their evolution. To achieve this, detailed molecular structural and functional information about both complexes is required – which, however, is not provided by Shimada et al (JBC, 2001). Although our present study

may indeed provide only the second structure of an animal mitochondrial aaRS-tRNA complex, it is the first to provide the opportunity to directly compare the molecular structural basis for specific interactions of two functionally related but structurally highly divergent mtDNA-encoded genes with a common nuclear-encoded binding partner. We therefore think that this study is in fact even more important for the understanding of animal mitochondrial biology than the previous one.

Furthermore, this reviewer is concerned about the biological relevance of the study as the authors use in vitro transcribed tRNAs, which lack post-transcriptional modifications. Even though the authors analyze in great detail the structure of mSerRS with tRNASer(UGA), they neglect the fact that some of the residues analyzed are post-transcriptionally modified in vivo. This mainly concerns the T-arm, where three modifications have been identified previously (Suzuki et al., Nat. Commun. 2020). The T-arm is a main contributor to the mSerRS interaction, and all modification sites seem to be participating in this interaction. Notably, these residues are highly conserved in the 41 primate species analyzed in the manuscript (Figure 3a), which supports their importance. Kuhle et al. compare this region structurally to its isoacceptor and identify differences, which are presented as major findings in the manuscript (Figure 3c and d, 4d, 5 a-c, 6a and b). Still, the potential effect of modifications is not considered.

Thank you for pointing out this omission. It is important to note that all three of these modifications in the T-loop of mtRNA$^{Ser(UGA)}$ ($m^5U54$, ψ55, and $m^1A58$) are part of the canonical repertoire of tRNA modifications and are thought to play important roles in stabilizing the canonical tRNA fold. Specifically, $m^5U54$ and $m^1A58$ form a reverse Hoogsteen pair that closes off the T-loop U-turn to the T-stem and ψ55 forms a conserved tertiary H-bond to G18 of the D-loop. Although two of these positions (U54, U55) indeed lie in the interface with mSerRS (A58 is not), these interactions are exclusively with the phosphate backbone and not with the bases. Thus, their most likely effect is to further stabilize the canonical conformation of the outer elbow region that we observe for mtRNA$^{Ser(UGA)}$ in our structure (consistent with analyses of the bovine mtRNA$^{Ser(UGA)}$ [Hayashi et al., JMB, 1998]) and therefore further manifest its structural differences to the non-canonical (and unmodified) T-loop of mtRNA$^{Ser(GCU)}$. As suggested by the reviewer, we included a discussion of post-transcriptional modifications in the revised manuscript on p. 6 line 112 and p. 12 line 239.

Other comments:

• In lines 177-179, the authors write:
“To bind mtRNASer(UGA), mSerRS involves 23 residues that are nearly identical to those involved in GCU isoacceptor binding.“

Yet, the authors write in lines 23+24:
“We find that despite their common function, mtRNASer(UGA) and mtRNASer(GCU) show no constrain to converge on a shared structure or sequence motif for interactions with mSerRS.”

Therefore, the similarities and differences of both binding modes are not clear. Are there any structural differences in the binding modes besides the T-arm region?

Thank you for raising this point, which appears to be a misunderstanding due to a lack of clarity on our part. What we tried to express is that although the synthetase uses almost the same 23 amino acid residues to bind both mtRNAs, the mtRNAs themselves do not converge on shared identity features (in structure or sequence). In other words, while the quote from lines 23-24 refers to the distinct **identity features of both mtRNAs** that provide identity information (i.e. the T-arm/elbow) for mSerRS, the second quote (lines 177-179) refers to the nearly identical 23 **amino acid side chains of mSerRS** involved in binding both tRNAs.
To clarify, the interactions with the acceptor stem are indeed the same for both mtRNASer isoacceptors, but they are not involved in determining the identities of the tRNAs. By contrast, the residues involved in T-arm binding are also the same, but as described on p. 10-11, the contributions of individual mSerRS residues are different for each $tRNA^{Ser}$ isoacceptor.
To improve the clarity of these passages, we changed them as follows:
Lines 23-24: “We find that despite their common function, mtRNASer(UGA) and mtRNASer(GCU) show no constrain to converge on a shared **structural or sequence identity motif for recognition by** mSerRS.”
Lines 177-179 (new count 189-190): “To bind $mtRNA^{Ser(UGA)}$, mSerRS **uses 23 amino acid residues** that are nearly identical to those involved in GCU isoacceptor binding.”

• In Shimada et al. (JBC 2001) it was demonstrated that mSerRS can misacylate mitochondrial tRNAGln due to the same sequence in the T-arm. It would be interesting to compare the structure of mtRNASer(UGA) and mtRNAGln bound to mSerRS or at least discuss it in the text.

We included a discussion of this point in the text on p. 11 line 218, on p. 12 line 243, and on p. 15 line 304.

**REVIEWERS' COMMENTS**

Reviewer #1 (Remarks to the Author):

Thank you for your responses to my comments, they are completely adequate.

Reviewer #3 (Remarks to the Author):

All points were addressed by the authors. This reviewer is satisfied with the revised manuscript.
